# Cancer/testis antigen CAGE mediates osimertinib resistance in non-small cell lung cancer cells and predicts poor prognosis in patients with pulmonary adenocarcinoma

**DOI:** 10.1038/s41598-023-43124-8

**Published:** 2023-09-21

**Authors:** Minjeong Yeon, Hankyu Lee, Jeongseon Yeo, Myeong Seon Jeong, Hyun Suk Jung, Hyerim Lee, Kyeonghee Shim, Hyein Jo, Doyong Jeon, Jaemoon Koh, Dooil Jeoung

**Affiliations:** 1 Department of Biochemistry, College of Natural Sciences, Kangwon National University, Chuncheon, South Korea; 2 L-Base Company, Seoul, South Korea; 3 Chuncheon Center, Korea Basic Science Institute, Chuncheon, Korea; 4 Department of Pathology, College of Medicine, Seoul National University, Seoul, Korea; 5 Present Address: The Wistar Institute, 3601 Spruce Street, Philadelphia, PA 19104 USA; 6 Present Address: Paean Biotech Company, Seoul, South Korea

**Keywords:** Biochemistry, Cancer, Molecular biology

## Abstract

CAGE, a cancer/testis antigen, was originally isolated from the sera of patients with gastric cancers. Previously, we have shown the role of CAGE in resistance to chemotherapy and target therapy. The aim of this study was to investigate the role of CAGE in osimertinib resistance and determine the prognostic value of CAGE in patients with pulmonary adenocarcinomas. The clinicopathological correlation with CAGE and autophagy flux in patients was examined using immunohistochemistry and in situ hybridization. The possible role of autophagy in osimertinib resistance was analyzed using immune blot, immune fluorescence staining and immunohistochemistry. This study found that immunohistochemical staining (IHC) showed CAGE expression in more than 50% of patients with pulmonary adenocarcinomas (pADCs). CAGE expression was increased in pADCs after the acquisition of EGFR-TKIs resistance. High expression of CAGE was correlated with shorter overall survival and progression free survival in patients with pADCs. Thus, CAGE mediates osimertinib resistance and predicts poor prognosis in patients with pADCs. Osimertinib-resistant non-small cell lung cancer cells (PC-9/OSI) were established and mechanistic studies of CAGE-mediated osimertinib resistance were performed. PC-9/OSI cells showed increased autophagic flux and CAGE expression compared with parental sensitive PC-9 cells. PC-9/OSI cells showed higher tumorigenic, metastatic, and angiogenic potential compared with parental PC-9 cells. CAGE CRISPR-Cas9 cell lines showed decreased autophagic flux, invasion, migration potential, and tumorigenic potential compared with PC-9/OSI cells in vitro and in vivo. CAGE plays a crucial role in the cancer progression by modulating autophagy and can predict the poor prognosis of patients with pulmonary adenocarcinomas. Our findings propose CAGE as a potential therapeutic target for developing anticancer drugs that can overcome osimertinib resistance.

## Introduction

Osimertinib, a third-generation epidermal growth factor receptor (EGFR) tyrosine kinase inhibitor (TKI), has been approved for the treatment of metastatic EGFR T790M mutation-positive non-small cell lung cancer (NSCLC) patients who are resistant to TKI therapy. The T790M mutation is found in over half of patients with progressive NSCLC following treatment with first-generation TKIs. Osimertinib is effective in NSCLC cells carrying the EGFR T790M mutation, although it eventually leads to resistance to osimertinib within approximately 1 year^1^.

The mechanisms of osimertinib resistance are diverse and not fully understood. Previous clinical studies have reported the underlying mechanisms involved in other EGFR mutations, C797S and L798I, which also prevent drug binding, bypassing of MET and ERBB2 signaling, or over activation of MAPK by KRAS or MEK mutation. However, the majority of patients acquired resistance by as yet unknown mechanisms^2^. A recent study showed that osiimertinib resistance is accompanied by enhanced autophagy and glycolysis. Class III phosphoinositide 3-kinase (PI3K) VPS34 is responsible for increased autophagy and glycolysis in NSCLC cells^3^. Osimertinib resistance is caused via epithelial mesenchymal transition^4^. Stemness promoted by the CXCL8 feedback loop leads to osimertinib resistance^5^. Overexpression of proto-oncogene mesenchymal epithelial transition (MET) leads to osimertinib resistance^6^. In addition, osimertinib activates interferon signaling, which in turn may induce osimertinib resistance^7^. Genetic depletion of RNA helicase DDX3X activates interferon signaling to induce antitumor activity^8^. Trichostatin A (TSA), an inhibitor of histone deacetylases, overcomes osimertinib resistance by decreasing the expression of bromodomain and extra-terminal proteins (BETs)^9^.

The cancer associated gene *(CAGE),* a cancer/testis gene, was initially discovered in the sera of patients diagnosed with gastric cancers^10^. CAGE was detected in the sera of 12% of patients with early-stage gastric cancer^11^ and in 7 of 13 (53.8%) patients with microsatellite instability-positive endometrial cancer^12^. CAGE enhances resistance to anticancer drugs via binding to glycogen synthase kinase 3β (GSK3β). CAGE enhances the self-renewal and tumorigenic potential of melanoma cells^13^. Interestingly, CAGE regulates autophagy and the response to anticancer drugs by binding with Beclin1 in non-small cell lung cancer cells with EGFR mutations using an in vitro model. These studies suggest the role of CAGE in osimertinib resistance.

Since CAGE mediates anticancer drug resistance, high expression of CAGE in patients with pulmonary adenocarcinomas was expected. Based on the fact that autophagy can confer anticancer drug resistance, we hypothesized that CAGE could be positively correlated with autophagic flux in patients with pulmonary adenocarcinomas. In this study, we showed the role of CAGE in osimertinib resistance in relation to autophagic flux. We also showed the prognostic value of CAGE in pulmonary adenocarcinomas.

## Results

### CAGE expression is positively correlated with clinicopathological features and autophagic flux and is increased in patients with pulmonary denocarcinoma

**Figure 1:**
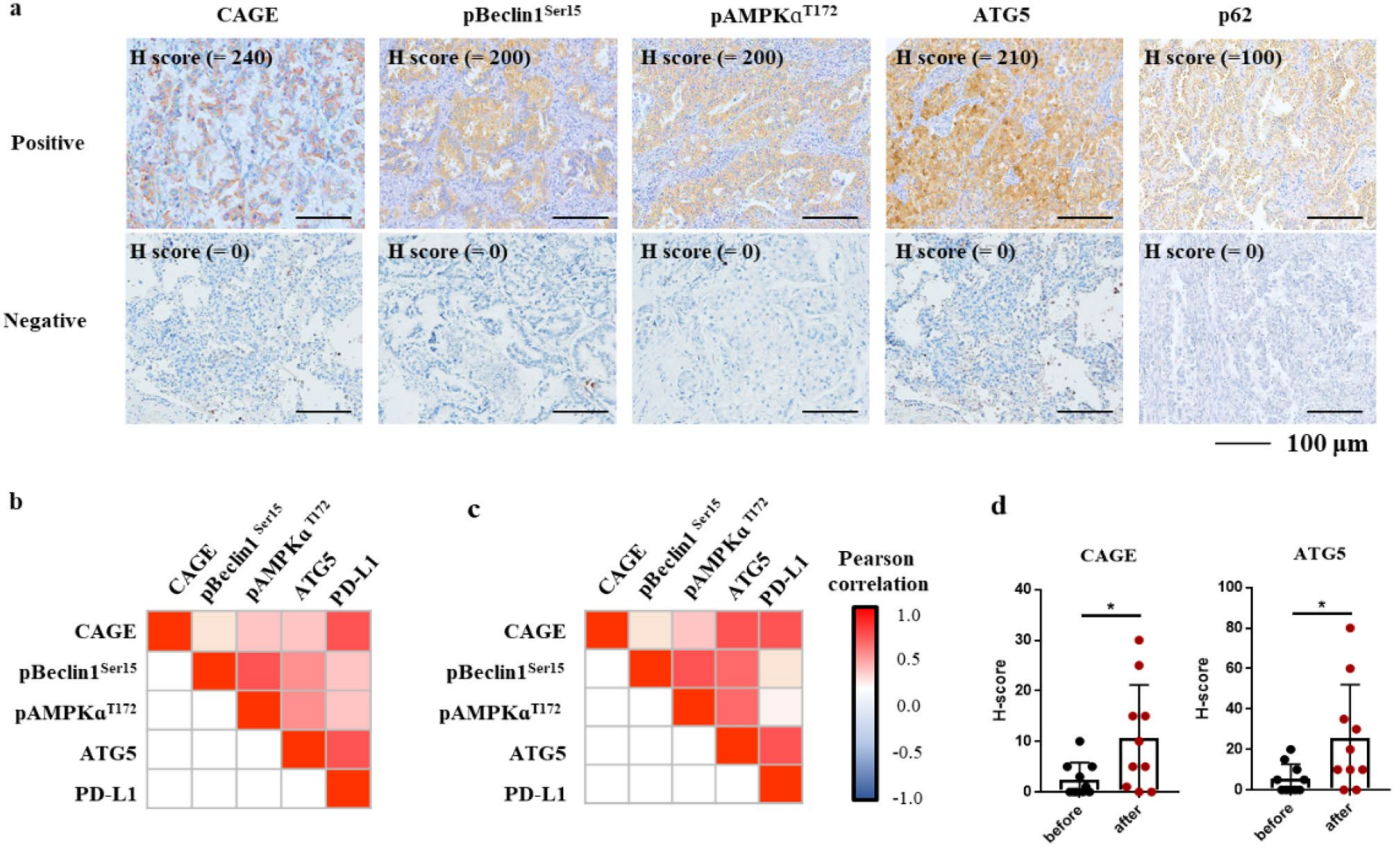
CAGE is increased after acquiring resistance to EGFR-TKIs resistance and is positively correlated with autophagy-related proteins. (**a**) Representative images of positive and negative expression of CAGE, pBeclin1Ser15, pAMPKɑT172, ATG5, and P62 in pulmonary adenocarcinomas (pADCs) (upper: positive expression, lower: negative expression; scale bar, 100 μm). (**b**) Correlations between CAGE and autophagy-related proteins in pADCs (Red, positive correlation; Blue, negative correlation). (**c**) Correlations between CAGE and autophagy-related proteins in EGFR mutated pADCs. Patients with pulmonary adenocarcinoma carrying EGFR mutations display exon 19 deletion and L858R in EGFR, but not T790M. (**d**) The H-scores of CAGE and ATG5 in paired tumor cells before and after acquisition of resistance to EGFR-TKIs. Paired biopsies were analyzed using the Wilcoxon Signed-Rank Test. *p < 0.05, Data were presented as the mean ± SEM).

Since CAGE mediates anticancer drug resistance^13^, this investigated the clinical relevance of CAGE. The expression of CAGE and autophagic flux in 220 patients with pADCs (Fig. 1a and Table 1) was analyzed by IHC. Among these 220 patients, 66 showed high CAGE expression with an H score over 40 (Table 1). The expression of pBeclin1Ser15, pAMPKɑT172 and ATG5 was detected in at least 30% of patients with pADCs (Table 1). CAGE expression was positively associated with autophagic flux including pBeclin1Ser15, pAMPKɑT172, and ATG5 but not P62 in patients with pADCs (Fig. 1b). In 77 cases of pADCs with EGFR mutations, CAGE expression was strongly correlated with, autophagic flux with statistical significance (Fig. 1c). Half of the patients (125/215) with pADCs carried EGFR mutations with exon 19 deletions or L858R. Paired tumor tissues from ten patients before EGFR-TKIs treatment and after acquiring resistance to EGFR-TKIs (gefitinib or erlotinib) were obtained. IHC revealed a significantly increased expression of CAGE and ATG5 in patients with pADC after the acquisition of EGFR-TKIs resistance (Fig. 1d). These results suggest that the increased expression of CAGE is strongly associated with EGFR-TKI resistance, in addition to autophagic flux, in patients with pADCs with or without EGFR mutations.

**Table 1:** Correlation between clinicopathological features and expression of CAGE, ATG5, pAMPKα^T172^, and pBeclin1^Ser15^ in pulmonary adenocarcinoma.

Molecular parameter		CAGE expression (40)			ATG5 expression (100)			pBeclin1^Ser15^ expression (10)			p-AMPKα ^T172^ expression (10)		
		Negative n (%)	Positive n (%)	*p*	Negative n (%)	Positive n (%)	*p*	Negative n (%)	Positive n (%)	*p*	Negative n (%)	Positive n (%)	*p*
Sex	Male	79 (75.2)	26 (24.8)	0.105	72 (72.0)	28 (28.0)	0.597	44 (42.7)	59 (57.3)	0.954	58 (56.9)	44 (43.1)	0.164
	Female	75 (65.2)	40 (34.8)		79 (68.7)	36 (31.3)		50 (43.1)	66 (56.9)		55 (47.4)	61 (52.6)	
Age	< 60	63 (68.5)	29 (31.5)	0.676	61 (68.5)	28 (31.5)	0.648	39 (41.5)	55 (58.5)	0.710	44 (47.3)	49 (52.7)	0.249
	≥ 60	91(71.1)	37 (28.9)		90 (71.4)	36 (28.6)		55 (44.0)	70 (56.0)		69 (55.2)	56 (44.8)	
Smoking	Never	86 (68.3)	40 (31.7)	0.436	89 (69.5)	39 (30.5)	0.687	54 (41.9)	75 (58.1)	0.678	65 (50.4)	64 (49.6)	0.582
	Ever	68 (73.1)	25 (26.9)		62 (72.1)	24 (27.9)		40 (44.9)	49 (55.1)		48 (54.5)	40 (45.5)	
Tumor size	< 3 cm	74 (69.8)	32 (30.2)	0.953	82 (79.6)	21 (20.4)	0.004	43 (40.6)	63 (59.4)	0.495	51 (48.1)	55 (51.9)	0.343
	≥ 3 cm	80 (70.2)	34 (29.8)		69 (61.6)	43 (38.4)		51 (45.1)	62 (54.9)		62 (55.4)	50 (44.6)	
LN metastasis	Negative	116 (73.0)	43 (27.0)	0.088	116 (75.3)	38 (24.7)	0.006	74 (46.8)	84 (53.2)	0.032	87 (55.4)	70 (44.6)	0.054
	Positive	36 (61.0)	23 (39.0)		33 (55.9)	26 (44.1)		18 (30.5)	41 (69.5)		24 (40.7)	35 (59.3)	
EGFR mutation	Absent	41 (71.9)	16 (28.1)	0.451	42 (75.0)	14 (25.0)	0.899	25 (45.5)	30 (54.5)	0.611	23 (42.6)	31 (57.4)	0.26
	Present	50 (65.8)	26 (34.2)		57 (74.0)	20 (26.0)		32 (41.0)	46 (59.0)		41 (52.6)	31 (47.4)	
EGFR expression	Negative	68 (69.4)	30 (30.6)	0.922	74 (77.1)	22 (22.9)	0.04	48 (50.0)	48 (50.0)	0.077	54 (56.8)	41 (43.2)	0.238
	Positive	84 (70.0)	36 (30.0)		75 (64.1)	42 (35.9)		46 (38.0)	75 (62.0)		59 (48.8)	62 (51.2)	
HER2 expression	Negative	150 (70.4)	63 (29.6)	0.431	148 (71.2)	60 (28.8)	0.2	93 (43.7)	120 (56.3)	0.241	112 (52.8)	100 (47.2)	0.080
	Positive	4 (57.1)	3 (42.9)		3 (42.9)	4 (57.1)		1 (16.7)	5 (83.3)		1 (16.7)	5 (83.3)	
c-MET expression	Negative	115 (72.8)	43 (27.2)	0.010	111 (71.2)	45 (28.8)	0.79	58 (47.2)	65 (52.8)	0.053	70 (57.4)	52 (42.6)	0.005
	Positive	23 (52.3)	21 (47.7)		29 (69.0)	13 (31.0)		26 (33.3)	52 (66.7)		29 (37.2)	49 (62.8)	
MET amplification	Absent	52 (69.3)	23 (30.7)	0.549	54 (73.0)	20 (27.0)	0.977	42 (56.0)	33 (44.0)	0.269	53 (70.7)	22 (29.3)	0.006
	Present	28 (63.6)	16 (36.4)		23 (72.7)	12 (27.3)		21 (45.7)	25 (54.3)		21 (45.7)	25 (54.3)	

Numbers in parentheses denote H scores. High expression of CAGE is defined by H scores greater than 40. High expression of ATG5 is indicated by H scores above 100. High expression of pBeclin1^Ser15^ and pAMPKα^T172^ is defined by H scores greater than 10.

This study analyzed the correlation between the clinicopathological features of pADCs with CAGE and autophagy. CAGE was more frequently expressed in patients with pADCs with lymph node metastasis and was positively correlated with the expression of c-MET (Table 1). ATG5 expression was positively associated with large tumor size, lymph node metastasis, and EGFR expression (Table 1). pBeclin1^Ser15^ expression correlated with lymph node metastasis in patients with pADCs (Table 1). pAMPKα^T172^ expression was positively correlated with c-MET expression, MET amplification, and lymph node metastasis (Table 1). In patients with pADCs carrying EGFR mutations, CAGE and ATG5 were more frequently expressed in those manifesting lymph node metastasis (Table 2). Thus, CAGE expression is positively correlated with lymph node metastasis and autophagic flux in patients with pADCs.

**Table 2:** Correlations between clinicopathological features and expressions of CAGE and ATG5 in pulmonary ADC with EGFR mutation.

Molecular parameter		CAGE expression (40)			ATG5 expression (100)		
		Negative n (%)	Positive n (%)	*p*	Negative n (%)	Positive n (%)	*p*
Sex	Male	14 (70.0)	6 (30.0)	0.644	16 (84.2)	3 (15.8)	0.368
	Female	36 (64.3)	20 (35.7)		41 (70.7)	17 (29.3)	
Age	< 60	20 (62.5)	12 (37.5)	0.606	23 (71.9)	9 (28.1)	0.717
	≥ 60	30 (68.2)	14 (31.8)		34 (75.6)	11 (24.4)	
Smoking	Never	34 (63.0)	20 (37.0)	0.275	41 (73.2)	15 (26.8)	0.765
	Ever	16 (76.2)	5 (23.8)		16 (80.0)	4 (20.0)	
Tumor size	< 3 cm	25 (67.6)	12 (32.4)	0.750	33 (86.8)	5 (13.2)	0.011
	≥ 3 cm	25 (64.1)	14 (35.9)		24 (61.5)	15 (38.5)	
LN metastasis	Negative	36 (73.5)	13 (26.5)	0.030	41 (83.7)	8 (16.3)	0.005
	Positive	12 (48.0)	13 (52.0)		14 (53.8)	12 (46.2)	
EGFR expression	Negative	24 (68.6)	11 (31.4)	0.637	30 (83.3)	6 (16.7)	0.081
	Positive	26 (63.4)	15 (36.6)		27 (65.9)	14 (34.1)	
HER2 expression	Negative	50 (66.7)	25 (33.3)	0.342	56 (73.7)	20 (26.3)	1.000
	Positive	0 (0.0)	1 (100.0)		1 (100.0)	0 (0.0)	
c-MET expression	Negative	36 (66.7)	18 (33.3)	0.492	41 (74.5)	14 (25.5)	0.604
	Positive	11 (57.9)	8 (42.1)		13 (68.4)	6 (31.6)	
MET amplification	Absent	20 (64.5)	11 (35.5)	0.848	26 (81.2)	6 (18.8)	0.736
	Present	13 (61.9)	8 (38.1)		16 (76.2)	5 (23.8)	

### High expression of CAGE predicts poor prognosis in patients with pulmonary adenocarcinomas

**Figure 2:**
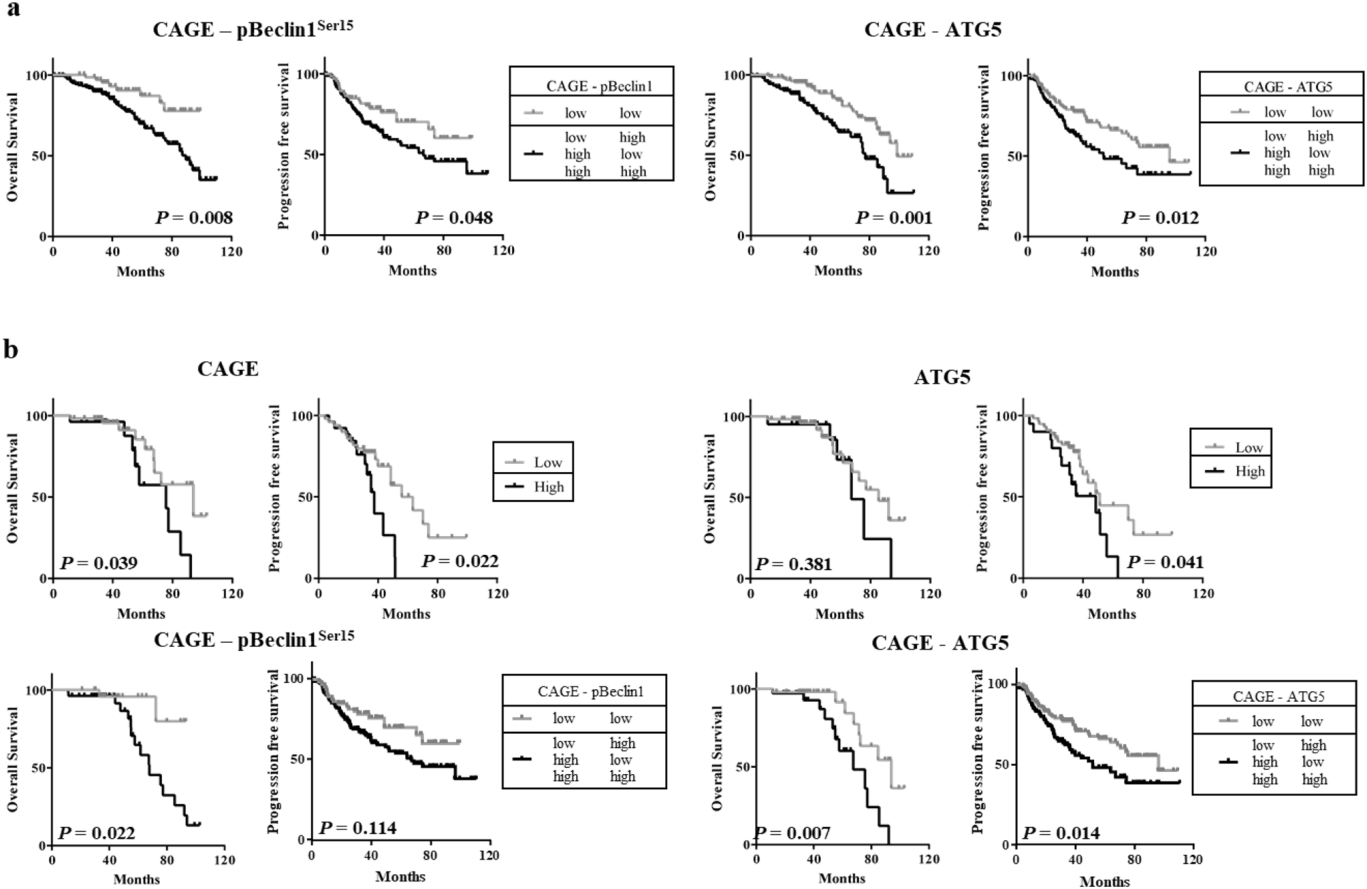
High expression of CAGE is correlated with poor prognosis in patients with pulmonary adenocarcinomas. (**a**) Kaplan–Meier plots with log-rank test for overall (OS) and progression-free survival (PFS) in patients with pADCs according to CAGE and pBeclin1Ser15 expression (left) and CAGE and ATG5 expression (right). H (high expression) represents H score greater than 40 for CAGE. In the case of pBeclin1^Ser15^, H denotes H score above 10. In the case of ATG5, H denotes H score higher than 100. (**b**) Kaplan–Meier plots with log-rank test for overall and progression-free survival in EGFR-mutated pADCs based on the expression of CAGE (left upper), ATG5 (right upper), CAGE and pBeclin1Ser15 (left lower) and CAGE and ATG5 (right lower). Patients diagnosed with pADCs carry EGFR mutations such as exon 19 deletion and L858R, but not T790M.

This study analyzed the effect of CAGE expression combined with pBeclin1^Ser15^ or ATG5 on the survival of patients with pADCs. Low expressions of CAGE and ATG5, or CAGE and pBeclin1^Ser15^ predicted high overall survival (OS) and progression-free survival (PFS) compared with high expression of CAGE, ATG5 or pBeclin1^Ser15^ (Fig. 2a). In patients with pADCs carrying the EGFR mutation, high expression of CAGE predicted poor OS and PFS compared with low expression of CAGE. High expression of ATG5 predicted shorter PFS, but not OS. Low expression of CAGE and pBeclin1^Ser15^ predicted higher OS compared with high expression of either protein. Low expression of CAGE and ATG5 predicted higher OS and PFS compared with high expression of either protein (Fig. 2b). Thus, CAGE alone or in combination autophagic flux can predict OS and PFS in patients with pADCs with or without EGFR mutation.

### CAGE regulates osimertinib-resistance, autophagic flux, and tumorigenic potential in non-small cell lung cancer cells

**Figure 3:**
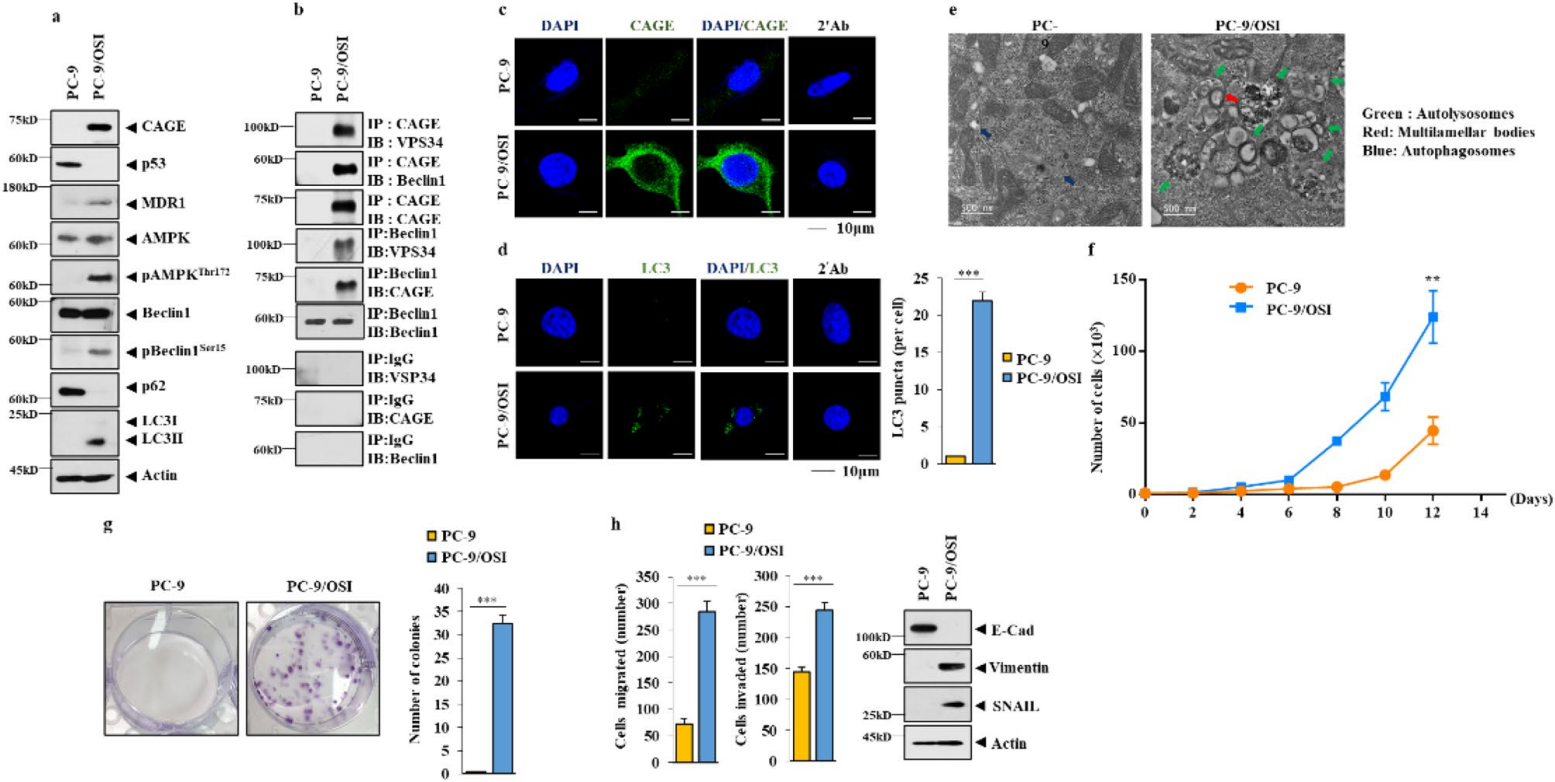
Osimertinib resistance is correlated with the enhanced autophagy, invasion, migration, and growth rates. Cell lysates from the indicated cancer cells were subjected to immunoblot (**a**) and immunoprecipitation (**b**). The uncropped blots are shown in Supplementary Materials. Immunofluorescence staining of CAGE (**c**) and LC3 (**d**) was performed. Representative images of three independent experiments were shown. Significance determined by one-way ANOVA. ***, *p* < 0.001. (**e**) Representative electron micrographs of PC-9 and PC-9/OSI cells were shown. The green arrows indicate autolysosomes. The red arrows indicate multivesicular bodies. (**f**) Cellular proliferation was determined by trypan blue exclusion assays. **, *p* < 0.01. (**g**) Colony forming potential was determined as described. Average values of three independent experiments were shown. Significance determined by one-way ANOVA. ***, *p* < 0.001. Cells were seed at 200 cells/dish. (**h**) Invasion and migration potential assays were performed. Average values of three independent experiments were shown. Immunoblot was also performed. Significance determined by one-way ANOVA. ***, *p* < 0.001. The uncropped blots are shown in Supplementary Materials.

Since CAGE showed correlations with clinicopathological features of patients with pADCs, it was interesting to examine the role of CAGE in anticancer drug resistance. Thus study aimed to investigate the potential role of CAGE in anticancer drug resistance in non-small cell lung cancer cells. For this, the sensitivity of these non-small cell lung cancer cell lines to osimertinib was determined. It was found that PC-9 cells were the most sensitive to osimertinib (Supplementary Fig. 1). Therefore, PC-9 cell were used to generate osimertinb-resistant PC-9 cells (PC-9/OSI). PC-9/OSI cells showed enhanced resistance to osimertinib (Supplementary Fig. 1 and Supplementary Table S3). In addition, PC-9/OSI cells exhibited increased expression of CAGE and autophagy-related factors such as pAMPKα^T172^, pBeclin1^Ser15^, and LC3II, but a decreased expression of p62 compared with parental sensitive cells (Fig. 3a). p62 is a selective receptor of autophagy. CAGE bound to Beclin1 and VPS34 (Fig. 3b) and was localized to the nuclear membrane and cytoplasm in PC-9/OSI cells (Fig. 3c). PC-9/OSI cells showed an increased number of LC3 puncta (Fig. 3d) and autophagosomes (Fig. 3e) compared with PC-9 cells. The growth rate of PC-9/OSI cells was increased compared with PC-9 cells (Fig. 3f,g). PC-9/OSI cells showed an enhanced migration and invasion potential compared with PC-9 cells (Fig. 3h). The expression of SNAIL and vimentin was increased in PC-9/OSI cells, whereas E-cadherin was decreased compared with PC-9 cells (Fig. 3h). These results imply that CAGE contributes to the pathogenesis of pADC by regulating autophagic flux and anticancer drug resistance.

Osimertinib resistance was closely associated with enhanced epithelial mesenchymal transition and autophagy^17^. Thus, the relationship between anticancer drug resistance and metastatic potential was examined. PC-9/OSI cells showed enhanced metastatic potential compared with PC-9 cells (Supplementary Fig. 2a). IHC (Supplementary Fig. 2b) and immunoblot (Supplementary Fig. 2c) of tumor tissues derived from PC-9/OSI cells showed increased expression of CAGE and autophagic flux compared with those derived from PC-9 cells. These data indicate that enhanced autophagic flux and anticancer drug resistance can contribute to the enhanced metastatic potential of cancer cells.

**Figure 4:**
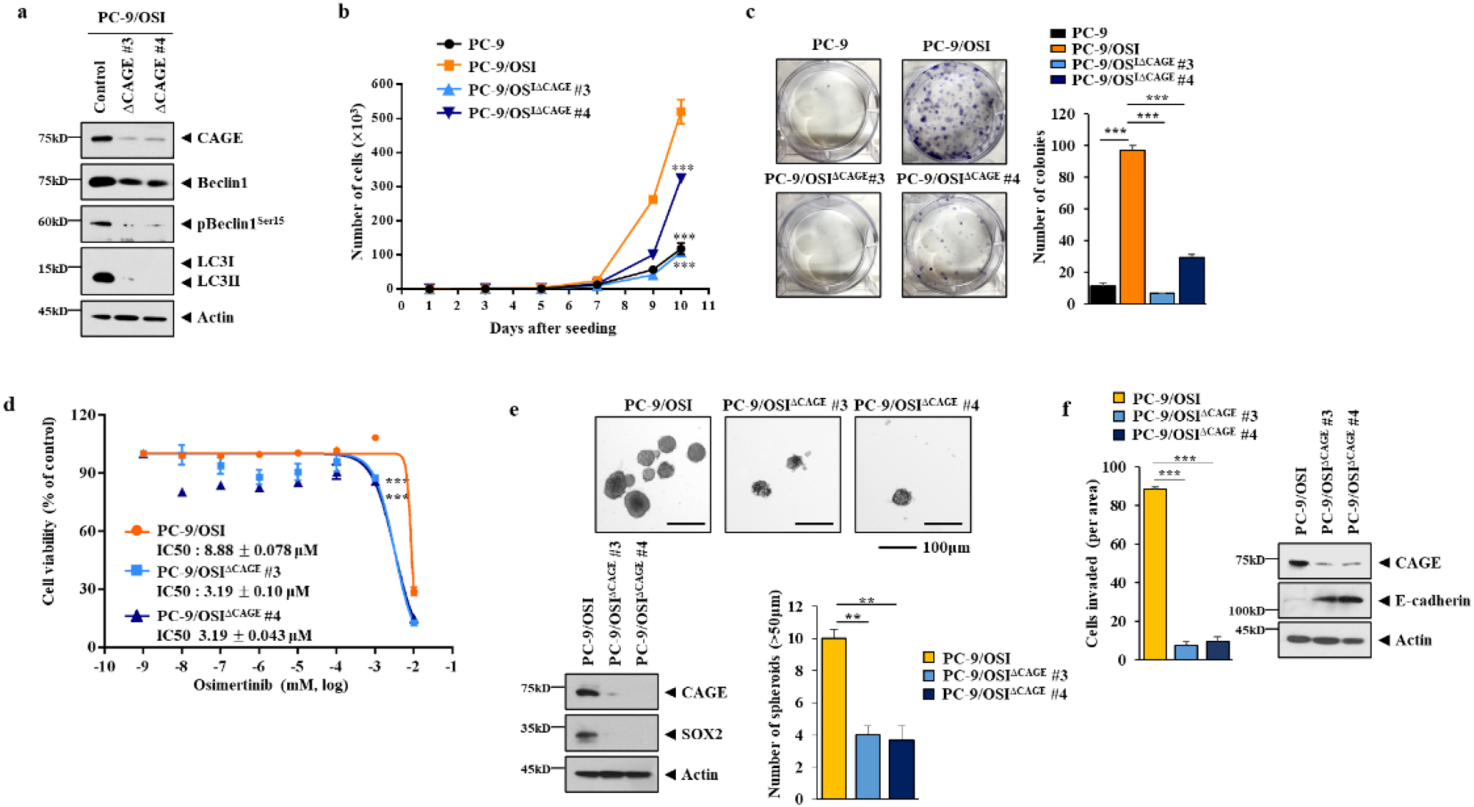
CAGE CRISPR-Cas9 cell lines show decreased autophagic flux and growth rates. (**a**) Cell lysates from the indicated cancer cell line were subjected to immunoblot. CAGE CRISPR-Cas9 cell lines were transfected with the indicated construct for 48 h, followed by immunoblot. The uncropped blots are shown in Supplementary Materials. (**b**) Cellular proliferation was determined by trypan blue exclusion assays. Significance determined by one-way ANOVA. ***, *p* < 0.001. (**c**) Colony forming potential of each cancer cell line was determined as described. Significance determined by one-way ANOVA. ***, *p* < 0.001. Cells were seed at 400 cells/dish. (**d**) Each indicated cell line was treated with various concentrations of osimertinib for 24 h. MTT assays were performed. Significance determined by one-way ANOVA. ***, *p* < 0.001. (**e**) Each CAGE CRISPR-Cas9 cell line was subjected to tumor spheroid forming potential assays. Immunoblot was also performed. Data are presented as mean ± SEM. Significance determined by one-way ANOVA. **, *p* < 0.01. The uncropped blots are shown in Supplementary Materials. (**f**) Invasion potential of the indicated cancer cell line was determined. Data are presented as mean ± SEM. Immunoblot was also performed. Significance determined by one-way ANOVA. ***, *p* < 0.001. The uncropped blots are shown in Supplementary Materials.

Next, the role of CAGE in tumorigenic potential was examined. For this, CRISPR/Cas-9 used to stably knock down CAGE expression in PC-9/OSI cells (PC-9/OSI^△CAGE#3^ and PC-9/OSI ^△CAGE#4^). PC-9/OSI^△CAGE#3^ and PC-9/OSI^△CAGE#4^ cell lines showed decreased autophagic flux compared with PC-9/OSI cells (Fig. 4a). PC-9/OSI^△CAGE#3^ and PC-9/OSI ^△CAGE#4^ cell lines showed decreased growth rates and colony forming potential compared with PC-9/OSI cells (Fig. 4b,c). PC-9/OSI^△CAGE#3^ and PC-9/OSI ^△CAGE#4^ cell lines showed enhanced sensitivity to osimertinib compared with PC-9/OSI cells (Fig. 4d). CAGE was necessary for tumor spheroid forming potential and the increased expression of SOX-2 PC-9 cells (Fig. 4e). PC-9/OSI^△CAGE#3^ and PC-9/OSI ^△CAGE#4^ cell lines showed decreased invasion potential compared with PC-9/OSI cells (Fig. 4f). These results indicate the role of CAGE in autophagic flux, growth rates, and invasion/migration of cancer cells.

Overexpression of CAGE increased autophagic flux (Supplementary Fig. 3a) and the number of LC-3 puncta (Supplementary Fig. 3b) in both PC-9/OSI^△CAGE#3^ and PC-9/OSI ^△CAGE#4^ cell lines. Overexpression of CAGE increased the tumor spheroid forming potential of PC-9/OSI ^△CAGE#4^ cell lines (Supplementary Fig. 3c). Overexpression of CAGE enhanced the invasion potential of the PC-9/OSI^△CAGE#3^ cell line (Supplementary Fig. 3d). These results confirm the role of CAGE in autophagic flux, growth rates, and invasion of cancer cells.

**Figure 5:**
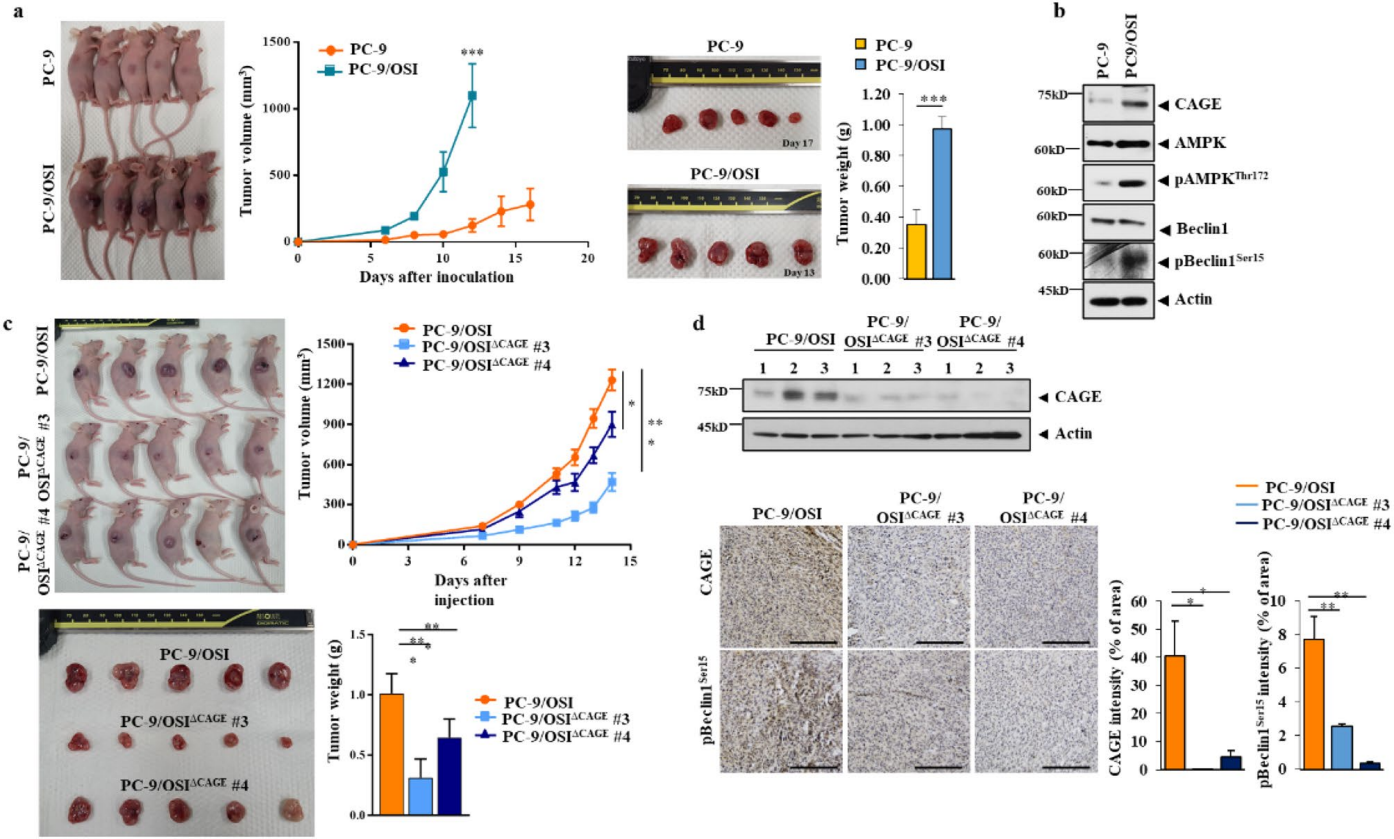
CAGE regulates tumorigenic potential in vivo. (**a**) The indicated cancer cells (each at 1 × 10^6^) were injected into the dorsal flanks of athymic nude mice. Tumor volumes were measured as described. Significance determined by one-way ANOVA.***, *p* < 0.001. Each experimental group consisted of five athymic nude mice. (**b**) Tumor tissue lysates were subjected to immunoblot. Representative blots of three independent experiments were shown. The uncropped blots are shown in Supplementary Materials. (**c**) The indicated cancer cells (each at 1 × 10^6^) were injected into the dorsal flanks of nude mice. Each experimental group consisted of five nude mice. Data are presented as a mean ± SEM. Tumor volumes were measured as described. Significance determined by one-way ANOVA. *, *p* < 0.05; ***, *p* < 0.001. (**d**) Tumor tissue lysates were subjected to immunoblot and immunohistochemical staining. Representative images of three independent experiments were shown. Data are presented as mean ± SEM. Significance determined by one-way ANOVA. *, *p* < 0.05; **, *p* < 0.01; ***, *p* < 0.001. The uncropped blots are shown in Supplementary Materials.

PC-9/OSI cells showed enhanced tumorigenic potential compared with PC-9 cells (Fig. 5a). This indicates that anticancer drug resistance is responsible for the enhanced tumorigenic potential. Tumor tissue lysates derived from PC-9/OSI cells showed increased expression of CAGE and pBeclin1^Ser15^ compared with those from PC-9 cells (Fig. 5b).

Both PC-9/OSI^△CAGE#3^ and PC-9/OSI^△CAGE#4^ cell lines showed low tumorigenic potential compared with PC-9/OSI cells (Fig. 5c). Immunoblot and IHC showed the expression of CAGE in tumor lysates derived from PC-9/OSI cells, but not those from PC-9/OSI^△CAGE#3^ or PC-9/OSI ^△CAGE#4^ cell lines (Fig. 5d). These results indicate that CAGE-promoted autophagy and anticancer drug resistance maybe responsible for the enhanced tumorigenic potential of PC-9/OSI cells.

## Discussion

The current therapeutic strategy requires novel approaches to overcome acquired resistance to various EGFR-TKIs, including osimertinib. Activation of autophagy flux induced resistance to EGFR-TKIs in non-small cell lung cancer cells^18^. Autophagy also promoted cellular survival under stress conditions in cancer^19^. Osimertinib resistance was correlated with enhanced autophagy and stem cell-like properties in EGFR-mutant NSCLC cells^17^. Thus, autophagy may serve as a target for overcoming resistance to EGRF-TKIs^20^. Previously, it was reported that CAGE, a cancer/testis antigen, enhanced the resistance of non-small cell lung cancer cells to erlotinib and gefitinib^21^. In this study, it was found that osimertinib resistance involved increased autophagy and CAGE expression in patients with pADCs. In addition, this study showed that high expression of CAGE predicted a poor prognosis and CAGE expression was positively correlated with lymph node metastasis and autophagy-related factors including ATG5, pBeclin1Ser15, and pAMPKɑT172 in patients with pADCS. Since autoantibodies against CAGE were found in the sera of patients with gastric cancers^11^, the serum level of CAGE in patients with pADCs needs to be determined.

It is known that CAGE promote cell proliferation, migration, invasion, and cellular stemness in various cancers. For example, CAGE promoted stem cell-like properties by upregulating the expression of stemness gene such as SOX2 in melanoma cells^22^. Furthermore, the expression of SOX2 and Nanog, a marker of cancer stemness, was increased in lung cancer cells resistant to gefitinib and osimertinib^23^. Cancer stem cells were also shown to enhance resistance to anticancer drugs in tongue cancer cells^24^. In this study, we showed the role of CAGE in conferring cancer stem cell-like properties. In addition, the study showed that CAGE was necessary for the enhanced tumorigenc potential of PC-9/OSI cells. CAGE was also necessary for the enhanced invasion/migration potential of PC-9/OSI cells.

The study showed that CAGE was responsible for the enhanced autophagic flux in osimertinib-resistant lung cancer cells and tumorigenic potential. The targeting of autophagy may be developed as a promising approach to overcome resistance to osimertinib. Some autophagy regulators, including chloroquine (CQ) and hydroxychloroquine (HCQ, CQ derivative), are used in cancer therapy. Both CQ and HCQ inhibit autophagic degradation and accumulation of autophagosomes. 3-Methyladenine(3-MA), an inhibitor of phosphatidylinositol 3-kinases (PI3K), blocks the formation of autophagosomes. CQ and 3-MA enhanced the sensitivity of glioblastoma cells to erlotinib^25^. Currently, clinical trials involving autophagy inhibitors have been initiated to assess therapeutic efficacies of autophagy inhibitors in cancer patients^26^. SB02024, a novel inhibitor of VPS34, can enhance the cytotoxicity of erlotinib on breast cancer cells^26^. However, almost all autophagy inhibitors have side effects involving gastrointestinal upset (vomiting and diarrhea), headache, skin rash, seizures, arrhythmia, bronchospasm, angioedema and dizziness^27^. Since protective autophagy is necessary for cell survival under stress, impaired autophagy has been linked to a variety of human diseases^28^.

In summary, this study showed that CAGE promoted protective autophagy and osimertinib resistance in NSCLCs. A positive correlation was found between CAGE expression and autophagic flux in patients with pulmonary lung adenocarcinomas. CRISPR-Cas9 knockdown of CAGE in osimertinib-resistant cell lines confirmed the role of the enhanced autophagic flux, tumorigenic potential, and metastatic potential of osimertinib-resistant lung cancer cells. Thus, CAGE can be used as a biomarker for the prediction of osimertinib resistance in patients with EGFR mutation. CAGE can also be a target for developing anticancer drugs that overcomes resistance to EGFR-TKIs, such as osimertinib.

## Materials and methods

### Materials

We purchased chemicals from Sigma Chemical Company. Anti- mouse and anti-rabbit IgG-horse radish peroxidase conjugate antibody were purchased from Pierce Company (Rockford, IL). Lipofectamine and PlusTM reagent for transfection were purchased from Invitrogen (San Diego, CA). Oligonucleotides, miRNA-mimic, and siRNAs used in this study were purchased from Bioneer Company (Daejon, Korea). Human recombinant CAGE protein was kindly provided by professor Dong Young Kim (Yeungnam University, Korea).

### Cell lines and cell culture

Cancer cell lines were cultured in Dulbecco’s modified minimal essential medium (Invitrogen) with 10% fetal bovine serum (Invitrogen). Cells were maintained at 37 °C in 5% CO_2_.

### Generation of osimertinib-resistant non-small cell lung cancer cell line

Osimertinib resistant non-small cell lung cancer cell line (PC-9/OSI) was generated by culturing PC-9 parental line with sequentially increasing concentrations of osimertinib^14^. Simultaneously, parental sensitive PC-9 cells were cultured in media containing DMSO (0.01%, Sigma-Aldrich) to generate control line (PC-9).

### Colony forming potential and cellular proliferation

Cells were seeded at a density of 100, 200, or 400 cells/35 mm dish. Colonies were stained with 0.01% crystal violet (Sigma-Aldrich; Merck KGaA). Cells were grown for 2 weeks until the colonies were formed.

MTT assays were employed to determine the response to anticancer drugs. Viable cell number was determined by trypan blue exclusion assays.

### Chemo invasion assays

Chemoinvasion assays were performed according to the standard procedures^15^. In brief, trypsinized cells (5 × 10^3^) in the serum-free RPMI 1640 medium were added to each upper chamber of the transwell chamber system with 8-μm pore polycarbonate filter inserts (CoSTAR, Acton, MA). RPMI 1640 medium supplemented with 10% fetal bovine serum was placed in the lower chamber and cells were incubated at 37 °C for 16 h. Cells remaining in the upper chamber were removed with a moist cotton swab. Cells that had migrated to the lower side of the chamber were fixed, stained with 0.1% crystal violet (Sigma-Aldrich, St Louis, MO, USA). and examined under a BZ-X800 all-inone microscope (Keyence Engineering Corporation). The number of cells that migrated to the lower side of the membrane was counted by using the ImageJ software. Differences were considered significant when *p* < 0.05.

### Tumor spheroid forming potential

Tumor spheroid forming potential assays were performed according to the standard procedures^15^. The total number of spheres was counted after 7 days by inverted microscopy (Olympus).

### Generation of knockout cell line with CRISPR/Cas9 system

Generation of CAGE (NCBI ENTREZ Gnee: 168400) knockout cell lines was performed according to the standard procedures^15^. In brief, CRISPR/Cas9-mediated gene editing was performed. A plasmid encoding Cas9 was purchased from Tool Gen. For sgRNA expression, the hU6-sgRNA plasmid that targeted CAGE (5′-AGGCTAATCCAAGAGACCTTGGG-3′) was used (Tool Gen). PC-9/OSI cells were transfected with Cas9, hU6-sgRNA, and hygromycin B-resistant reporter plasmid (ToolGen). After 48 h of transfection, cells were treated with hygromycin B (150 μg/ml) three times a week. Hygromycin-resistant colonies were isolated and subjected to immunoblot. Mismatch sensitive nuclease assays was performed to validate correctness of CAGE knockout.

### Immunofluorescence staining

Cells were washed and fixed with 4% paraformaldehyde before permeabilization with Triton X-100. After blocked with goat serum (10%) in 0.1% BSA/PBS, cells were incubated with anti-LC3 or anti-CAGE at 4 °C overnight and then incubated with anti-rabbit Alexa Fluor 488 secondary antibody. After removal of antibodies, cells were stained with DAPI and mounted with mounting medium. The immune fluorescent images were observed and captured using a confocal laser scanning microscopy (Nikon, Eclipse TS100, phase contrast fluorescence microscope). Images were analyzed by NIS-Elements AR software.

### Immunoblot and immunoprecipitation

Immunoblot was performed according to the standard procedures^13^. To isolate tissue lysates, tissue was frozen in liquid nitrogen and homogenized using lysis buffer. After vortexing and centrifugation at 10,000 × g for 15 min at 4 °C, supernatant was used as tissue lysates. Lysates (20 µg/well) were separated through 10% SDS-PAGE, followed by transfer onto PVDF membrane. Subsequently, membrane was immersed within BSA (2% w/v) for a 2 h, followed by overnight incubation under 4 °C using corresponding primary antibodies. The following primary antibodies were used in this study: CAGE (1:1000, MBS2524843; MyBioSource); AMPKα (1:100, AF3194; R&D Systems); pAMPKα^*Thr172*^ (1:1000, 2535S; Cell Signaling), PARP (1:1000, 9542S; Cell Signaling), pBeclin1^*Ser15*^ (1:1000, 84966S; Cell Signaling), LC3 (1:1000, 12741S; Cell Signaling), Bcl-2 (1:1000, 3498S; Cell Signaling), E-cadherin (1:1000, 3195S; Cell Signaling), Vimentin (1:1000, 5741S; Cell Signaling), mTOR (1:1000, 2972S; Cell Signaling), pmTOR^*Ser*2448^ (1:1000, 2971S; Cell Signaling), Alix (1:1000, 2171S; Cell Signaling), p53 (1:1000, 2524S; Cell Signaling), Beclin1 (1:1000, sc-48341; Santa Cruz), IgG (1:1000, sc-2025; Santa Cruz), SNAIL (1:1000, sc-271977; Santa Cruz), ATG5 (1:2000, sc-133158; Santa Cruz), PAI-1 (1:1000, sc-5297; Santa Cruz), Biotin (1:1000, sc-101339; Santa Cruz), TSG101 (1:1000, sc-7964; Santa Cruz), CD81 (1:5000, sc-166029; Santa Cruz), Actin (1:2000, A2228; Sigma), FLAG (F3166,;Sigma), Caspase-3 (PA05689A0Rb; Cusabio), p62 (1:1000, ab56416; Abcam), MDR1 (1:1000, CSB-PA1173A0; Cusabio), and S1PR1 (1:3000, 55133-I-AP; Proteintech). The following secondary antibodies were used in this study: anti-mouse HRP secondary antibody (31430, Invitrogen), anti-goat HRP secondary antibody (31402, Invitrogen), anti-rabbit HRP secondary antibody (ADI-SAB-300-J, Enzo), and anti-rabbit Alexa Fluor 488 secondary antibody (A11008, Invitrogen). For immunoprecipitations, cell lysates or tissue lysates (100–200 μg) were immunoprecipitated with respective primary antibody (0.2–2 μg) for overnight at 4 °C. Twenty μl of Protein A/G PLUS-Agarose (Santa Cruz) was then added and incubation was continued for 1 h at 4 °C. Beads were washed three times with lysis buffer, 2X sample buffer was added. Samples were then denatured (100 °C for 5 min) and analyzed 10% SDS-PAGE, followed by immunoblot. Band intensity was quantified using Image-J. The detailed information of primary antibodies is described in Supplementary Table S1.

Some blots were cut prior to hybridization with antibodies.

### Electron microscopic observation of autophagic process

Cells were treated with the fixing solution (2.5% glutaraldehyde in 0.1 M cacodylate solution (pH 7.0) for 1 h), and then mixed with 2% osmium tetroxide for 2 h at 4 °C. The samples were dehydrated with a graded acetone series, and embedded into Spurr medium (Electron Microscopy System). The samples were sectioned (60 nm) by using ultra-microtome (RMC MTXL, Arizona, USA). The section was stained with 2% uranyl acetate for 20 min followed by staining with lead citrate for 10 min. The sections were then viewed under a transmission electron microscope (JEM-2100F, Japan) at 200 kV.

### In vivo tumorigenic potential

Cancer cells (1 × 10^6^) were injected subcutaneously into the dorsal flank area of the BALB/c mice to induce formation of tumors. All animal experiments were performed according to the guidelines of the Korean Council for the Care and Use of Animals in Research and approved by the Institutional Animal Care and Use Committee (IACUC) of Kangwon National University and are in compliance with ARRIVE guidelines. All experiments were carried out in accordance with approved guidelines and regulations. Tumor volume (0.5 × length × width^2^) was calculated. For in vivo metastasis assay, PC-9 or PC-9/OSI cells (1 × 10^6^ cells in PBS) were injected intravenously into the tail vein of 4-week-old female athymic nude mice, and the extent of lung metastasis was evaluated. Mice were first anesthetized with intraperitoneal injection of 250 mg/kg tribromoethanol (Avertin, Sigma-Aldrich, USA). Animal euthanasia was performed using CO_2_ gas at 30–70% displacement rate of the cage volume/min using a flow meter according to the American Veterinary Medical Association (AVMA) euthanasia guideline43.

### Immunohistochemical staining (IHC) of tumor tissue

Sections of the paraffin-embedded tissue blocks (4–6 μm-thick) were mounted on positively charged glass slides, and dried in an oven at 56 °C for 30 min. The sections were deparaffinized and then rehydrated, and hydrogen peroxide was added to suppress endogenous peroxidase. After treatment with bovine serum albumin (BSA) to block nonspecific binding, the sections were then incubated with primary antibody overnight at 4 °C. After washing, biotinylated secondary antibody was added for 1 h. Diaminobenzidine (Vector Laboratories, Inc.) was employed for color development. Mayer’s hematoxylin was used for counterstaining of sections. The detailed information of primary antibodies is described in Supplementary Table S1.

### Statistical analysis

Statistical analysis was performed using the GraphPad Prism statistics program (Version 7). All the data were obtained from experiments with adequate sample size and presented as means ± SEM. One–way ANOVA was carried out for comparisons among three or more groups and was followed by Tukey’s post hoc test. Values were considered to be significant at *p* < 0.05. Student’s t-test was also employed.

### Patients and samples

We collected tissues from 215 patients with pulmonary adenocarcinoma who underwent surgery and had been followed up at Seoul National University Hospital (Seoul, Republic of Korea) from 2001 to 2011. None had received chemotherapy before surgery or had distant metastasis at the time of diagnosis. Clinicopathologic data and pathologic tumor–node– metastasis (TNM) staging from the 8th American Joint Committee on Cancer were obtained from medical and pathologic records. Formalin-fixed paraffin-embedded (FFPE) lung non-small cell lung cancer tissue that had been stored as a formalin fixed paraffin embedded block was obtained. A tissue microarray was constructed from 2-mm diameter cores derived from representative tumor areas of formalin-fixed, paraffin embedded tissue blocks. EGFR, KRAS mutation and ALK translocation status were evaluated as described previously^16^. FFPE of TMA blocks were cooled to − 5 °C on ice blocks and serially sectioned using a microtome (RM2125 RTS, Leica Biosystems, Germany) at 4 μm thickness. Sections were mounted onto charged glass slides (Superfrost Plus Slides, Thermo Fisher Scientific, Carlsbad, CA) and baked in a drying oven at 52 °C for 30 min. An Autostainer XL (ST5010, Leica Biosystems, Nussloch, Germany) was used to perform standard H&E staining. Another cohort of 10 patients who had recurrence or metastatic pADCs with EGFR mutation and were treated with the EGFR-TKI at SNUH was collected for evaluation of CAGE and ATG5 expression. PFS was measured from the first day of inhibitor treatment until the first objective sign of disease progression or death. OS was measured from the date of diagnosis until death from any cause. This study followed the World Medical Association Declaration of Helsinki recommendations and was approved by the Institutional Review Board of Seoul National University Hospital (H-1404-100-572).

### IHC and fluorescence in situ hybridization

Immunohistochemistry of CAGE (1:100, MBS626876, MyBioSource), ATG5 (1:400, 66744-v1-Ig, Proteintech), pBeclin1Ser15 (1:50, AF2323, Affinity biosciences), pAMPKɑT172 (1:200, 40H9, Cell Signaling Technology) or p62 (1:200, D5E2, Cell Signaling Technology) was performed using the Benchmark XT autostainer (Ventana Medical Systems). Whole-slide images were obtained by the Aperio ScanScope slide scanner (Aperio Technologies, Vista, CA, USA). All the cases were scanned on the Aperio AT2 scanner at × 40 (0.23 um/pixel). The CAGE, ATG5, pBeclin1^Ser15^, pAMPKɑ^T172^ and p62 immunohistochemistry were evaluated based on the intensity and proportion of cytoplasmic or nuclear staining in tumor cells and the results (H-score) are recorded by multiplying the percentage of positive cells by the intensity. Immunohistochemistry for EGFR and MET was assessed by modified criteria of previous report. Cases were scored into 0 (absent or only focal weak), 1 (weak to moderate in ≤ 40% of tumor cells), 2 (weak to moderate in ≥ 40% of tumor cells), and 3 (strong in ≥ 10% of tumor cells); and then classified into negative (score 0 or 1) and positive (score 2 or 3) for EGFR and MET expression. HER2 expression was scored from 0 to 3 + according to the FDA approved guidelines for the HercepTest and cases with scores of 2 + or 3 + were considered positive for expression. The list of antibodies used in IHC is described in supplementary Table S2.

MET GCN and amplification was estimated using and LSI MET Spectrum Red/CEP7 Spectrum Green probe (Abbott Molecular, DesPlaines, IL, USA), and was counted in at least 100 tumor nuclei. Gene amplification (MET to CEP7 ratio ≥ 2; > 15 copies of the MET signals in > 10% of the tumor cells; small gene cluster [4–10 copies] or innumerable tight gene cluster in > 10% of the tumor cells) and high polysomy (≥ 40% of cells displaying ≥ 4 copies of the MET signal) were defined as FISH positivity according to University of Colorado Cancer Center (UCCC) criteria^16^.

### Statistical analyses of human samples

All statistical analyses were performed using SPSS software (version 25; SPSS, Chicago, IL, USA). Comparisons between variables were performed using the χ^2^ test, Fisher’s exact test, or Student’s t-test. Cut-off values of continuous variables were determined based on the receiver operating characteristic curve at the highest positive likelihood point for disease-free survival. Survival analysis was performed using the Kaplan–Meier method with log-rank test. Two-sided *p* values of < 0.05 were considered statistically significant.

### Ethics approval and consent to participate

All authors approved and directly participated in the planning, execution and/or analysis of the data presented here. All protocols were approved by the Institutional Review Board of SNUH (H-1404-100-572) and all participants provided written informed consent. All experiments including animal were in accordance with by Institutional Animal Care and Use Committee (IACUC) of Kangwon National University (KW-200803-1).

### Supplementary Information

Supplementary Information 1.

Supplementary Information 2.

Supplementary Information 3.

## Data Availability

Correspondence and requests for materials should be addressed to D.J. D.J. takes responsibility for the integrity of the data and the accuracy of the data analysis. The datasets used during the study are available from the corresponding author (D.J.) upon reasonable request.
