# Supplementary material for: Cancer/testis antigen CAGE mediates osimertinib resistance in non-small cell lung cancer cells and predicts poor prognosis in patients with pulmonary adenocarcinoma
Source: Sci Rep. 2023 Sep 21;13:15748. doi: 10.1038/s41598-023-43124-8 (PMC10514060; doi:10.1038/s41598-023-43124-8)
Supplement: Supplementary file 1 — Supplementary Information 1. [file 41598_2023_43124_MOESM1_ESM.pdf]

SUPPLEMENTARY FIGURES

Title: Cancer/testis antigen CAGE mediates Osimertinib Resistance in Non-small cell lung cancer cells and predicts poor Prognosis in Patients with Pulmonary adenocarcinoma

Author list: Minjeong Yeon[1†], Hankyu Lee [2†], Jeongseon Yeo [1#], Myeong Seon Jeong[1, 3], Hyun Suk Jung[1], Hyerim Lee[2], Kyeonghee Shim[1], Hyein Jo[1], Doyong Jeon[2*], Jaemoon Koh[4*] and Dooil Jeoung[1*]

Affiliations:

[1] Department of Biochemistry, College of Natural Sciences, Kangwon National University, Chuncheon, Korea
[2] L-Base Company, Seoul. South Korea
[3] Chuncheon Center, Korea Basic Science Institute, Chuncheon, Korea
[4] Department of Pathology, College of Medicine, Seoul National University, Seoul, Korea

[*] Correspondence: Dooil Jeoung
jeoungd@kangwon.ac.kr
Jaemoon Koh
66020@snuh.org
Doyong Jeon
David.jeon@l-base.com

[+]these authors contributed equally to this work
1† Present address: The Wistar Institute, 3601 Spruce Street, Philadelphia, PA 19104
1# Present address: Paean Biotech Company, Seoul, South Korea

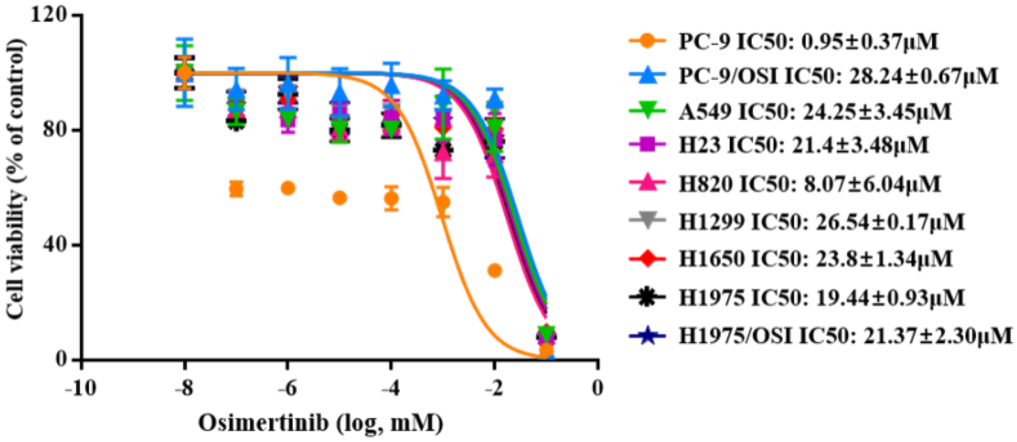

Supplementary Fig. 1 Establishment of Osimertinib resistant cell line.
The indicated cancer cell line was treated with various concentrations of osimertinib for 48 hours. MTT assays were performed. The IC_{50} value of each cell line was determined from the concentration-response curves. Data are presented as mean ± SEM.

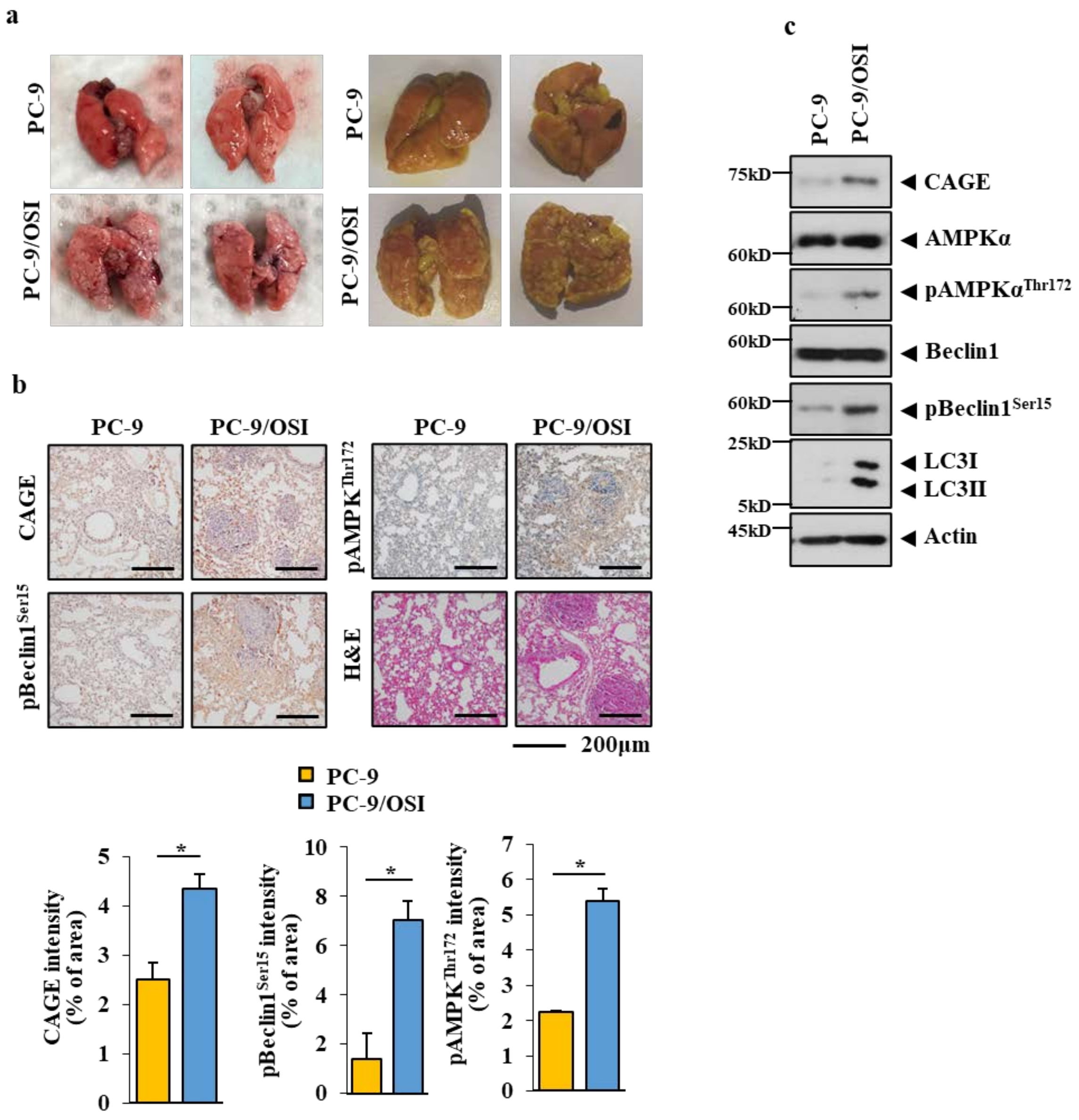

Supplementary Fig. 2 Osimertinib resistance is correlated with enhanced metastatic potentials and autophagic flux. (**a**) The indicated cancer cells (1×10^6 cells in PBS) were injected intravenously into the tail vein of 4-week-old athymic nude mice, and the extent of lung metastasis was evaluated. Each experimental group consisted of five athymic nude mice. (**b**) Tumor tissues were subjected to immunohistochemical staining. Significance determined by one-way ANOVA. *, $p < 0.05$. Representative images of three independent experiments were shown. (**c**) Tumor tissue lysates were subjected to immunoblot. The uncropped blots are shown in Supplementary Materials.

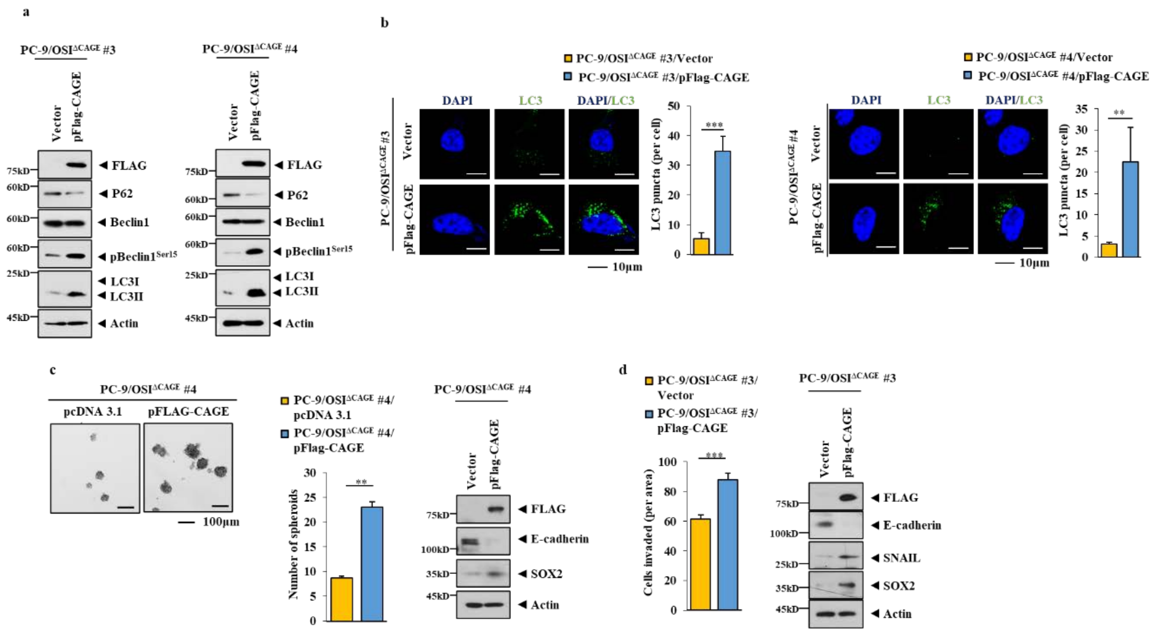

Supplementary Fig. 3 CAGE increases invasion, autophagic flux, and tumor spheroid forming potential of CAGE CRISPR-Cas9 cell lines. **(a)** CAGE CRISPR-Cas9 cell lines were transfected with the indicated construct for 48 h, followed by immunoblot. The uncropped blots are shown in Supplementary Materials. **(b)** Each CAGE CRISPR-Cas9 cell line was transfected with the indicated construct for 48 h, followed by immunofluorescence staining. Representative images of three independent experiments were shown. Data are presented as mean ± SEM. Significance determined by Student's t-test. **, *p<0.01*; ***, *p<0.001*. **(c)** CAGE CRISPR-Cas9 cell line was transfected with the indicated construct for 48 h, followed by tumor spheroid forming potential assays. Immunoblot was also performed (right). Data are presented as mean ± SEM. Significance determined by Student's t-test. **, *p<0.01*. **(d)** CAGE CRISPR-Cas9 cell line was transfected with the indicated construct for 48 h, followed by invasion assays. Immunoblot was also performed (right). Data are presented as mean ± SEM. Significance determined by Student's t-test. ***, *p<0.001*. The uncropped blots are shown in Supplementary Materials.
