# Supplementary material for: Cancer/testis antigen CAGE mediates osimertinib resistance in non-small cell lung cancer cells and predicts poor prognosis in patients with pulmonary adenocarcinoma
Source: Sci Rep. 2023 Sep 21;13:15748. doi: 10.1038/s41598-023-43124-8 (PMC10514060; doi:10.1038/s41598-023-43124-8)
Supplement: Supplementary file 2 — Supplementary Information 2. [file 41598_2023_43124_MOESM2_ESM.pdf]

SUPPLEMENTARY TABLES

Title: Cancer/testis antigen CAGE mediates Osimertinib Resistance in Non-small cell lung cancer cells and predicts poor Prognosis in Patients with Pulmonary adenocarcinoma

Author list: Minjeong Yeon[1†], Hankyu Lee [2†], Jeongseon Yeo [1#], Myeong Seon Jeong[1, 3], Hyun Suk Jung[1], Hyerim Lee[2], Kyeonghee Shim[1], Hyein Jo[1], Doyong Jeon[2*], Jaemoon Koh[4*] and Dooil Jeoung[1*]

Affiliations:
[1] Department of Biochemistry, College of Natural Sciences, Kangwon National University, Chuncheon, Korea
[2] L-Base Company, Seoul. South Korea
[3] Chuncheon Center, Korea Basic Science Institute, Chuncheon, Korea
[4] Department of Pathology, College of Medicine, Seoul National University, Seoul, Korea
[*] Correspondence: Dooil Jeoung
jeoungd@kangwon.ac.kr
Jaemoon Koh
66020@snuh.org
Doyong Jeon
David.jeon@l-base.com
[+]these authors contributed equally to this work
1† Present address: The Wistar Institute, 3601 Spruce Street, Philadelphia, PA 19104
1# Present address: Paean Biotech Company, Seoul, South Korea

Supplementary Table S1. List of antibodies used in immunoblot and immunoprecipitation.

Antibody	Supplier	Cat.
CAGE	Mybiosource	MBS2524843
AMPKα	R&D system	AF3194
pAMPKα^{T172}	Cell Signaling	2535S
PARP	Cell Signaling	9542S
pBeclin1^{Ser15}	Cell Signaling	84966S
LC3	Cell Signaling	12741S
E-Cadherin	Cell Signaling	3195S
Vimentin	Cell Signaling	5741S
Alix	Cell Signaling	2171S
VSP34	Cell Signaling	4263S
p53	Cell Signaling	2524S
Beclin1	Santa Cruz	sc-48341
IgG	Santa Cruz	sc-2025
SNAIL	Santa Cruz	sc-271977
ATG5	Santa Cruz	sc-133158
TSG101	Santa Cruz	sc-7964
SOX2	Santa Cruz	sc-398254
CD81	Santa Cruz	sc-166029
Actin	Sigma	A2228
FLAG	Sigma	F3166
p62	Abcam	ab56416
S1PR1	Proteintech	55133-I-AP
MDR1	Cusabio	CSB-PA1173A0

Supplementary Table S2. List of antibodies used in IHC.

Antibody	Vendor	Clone	Host, clonality	Final Dilution
CAGE	MyBioSource	#MBS9401569	Rabbit, polyclonal	1:100
ATG5	Proteintech	10181-2-AP	Rabbit, polyclonal	1:400
pBeclin1^{Ser15}	Affinity biosciences	AF2323	Rabbit, polyclonal	1:50
phospho AMPKα^{T172}	Cell Siganling Technology	40H9	Rabbit, monoclonal	1:200
p62	Proteintech	18420-1-AP	Rabbit, polyclonal	1:500
HER2	Santa Cruz Biotechnology	sc-33684	Mouse, monoclonal	1:200
c-MET	Ventana Medical Systems	SP44	Rabbit, monoclonal	Predilution
EGFR	Ventana Medical Systems	3C6	Mouse, monoclonal	Predilution
PD-L1	Dako	22C3	Mouse, monoclonal	pharmDx kit

Supplementary Table S3. Anti-cancer drug-resistance of PC-9 and PC-9/OSI cells.

IC50 (μM)		
	**PC-9**	**PC-9/OSI**
Osimertinib	0.72	3.56
Erlotinib	7.62	2.39E+20
Paclitaxel	0.02	3.06
Gefitinib	0.012	7.71

Note: The indicated cancer cell line was treated with the indicated anti-cancer drugs for 48 hours. The cell viability was analyzed by an MTT assay. The IC_{50} value of each cell line was determined from the concentration-response curves. All values indicate the mean ± SEM.
