# Supplementary material for: Cancer/testis antigen CAGE mediates osimertinib resistance in non-small cell lung cancer cells and predicts poor prognosis in patients with pulmonary adenocarcinoma
Source: Sci Rep. 2023 Sep 21;13:15748. doi: 10.1038/s41598-023-43124-8 (PMC10514060; doi:10.1038/s41598-023-43124-8)

RELATED FILE
Western Blot Data Uncropped Gel

Title: Cancer/testis antigen CAGE mediates Osimertinib Resistance in Non-small cell lung cancer cells and predicts poor Prognosis in Patients with Pulmonary adenocarcinoma

Author list: Minjeong Yeon[1†], Hankyu Lee [2†], Jeongseon Yeo [1#], Myeong Seon Jeong[1, 3], Hyun Suk Jung[1], Hyerim Lee[2], Kyeonghee Shim[1], Hyein Jo[1], Doyong Jeon[2*], Jaemoon Koh[4*] and Dooil Jeoung[1*]

Affiliations:

[1] Department of Biochemistry, College of Natural Sciences, Kangwon National University, Chuncheon, Korea
[2] L-Base Company, Seoul. South Korea
[3] Chuncheon Center, Korea Basic Science Institute, Chuncheon, Korea
[4] Department of Pathology, College of Medicine, Seoul National University, Seoul, Korea

[*] Correspondence: Dooil Jeoung
jeoungd@kangwon.ac.kr
Jaemoon Koh
66020@snuh.org
Doyong Jeon
David.jeon@l-base.com

[+]these authors contributed equally to this work
1† Present address: The Wistar Institute, 3601 Spruce Street, Philadelphia, PA 19104
1# Present address: Paean Biotech Company, Seoul, South Korea

Figure3a

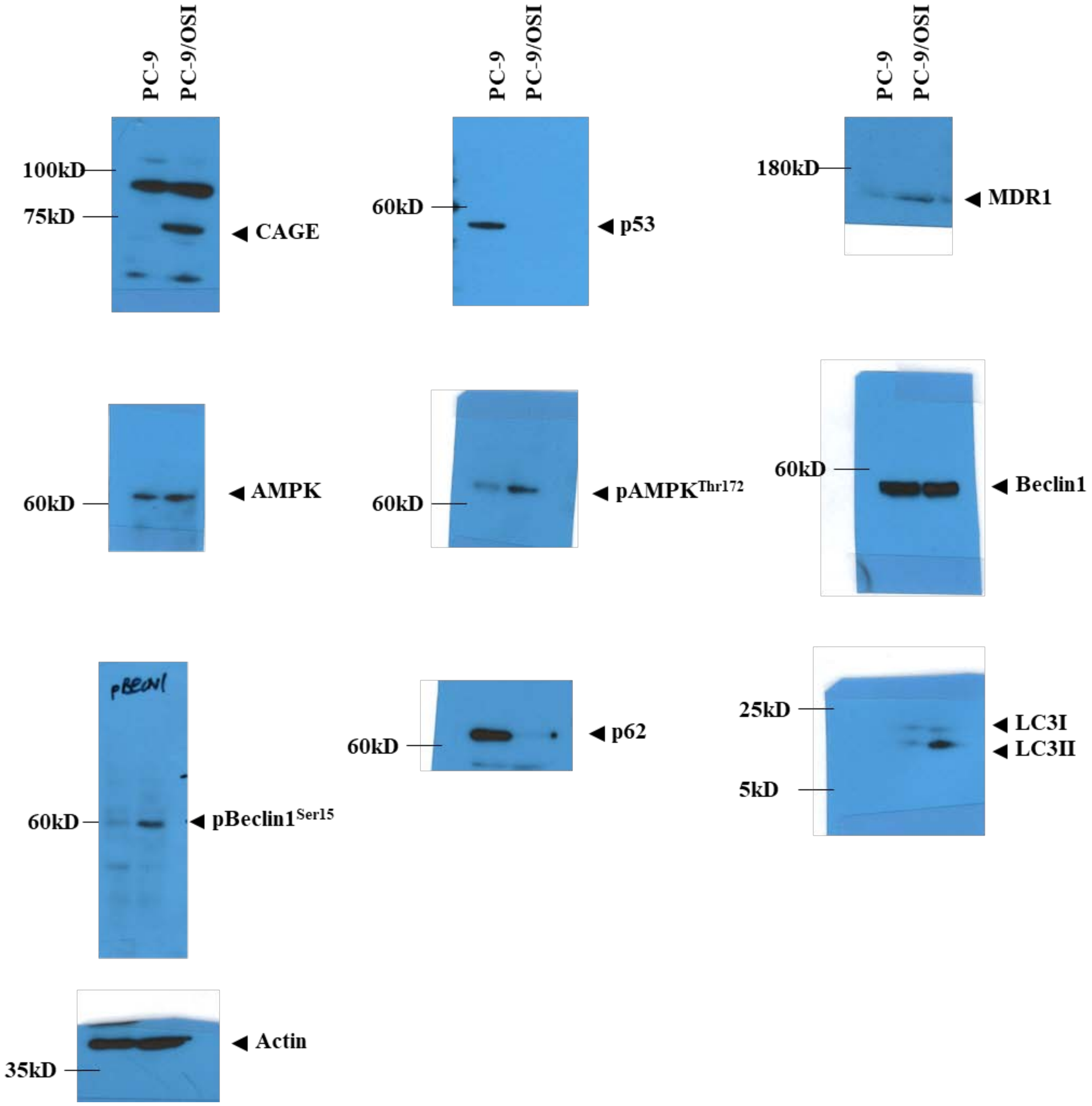
PC-9
PC-9/OSI
100kD
75kD
CAGE
60kD
p53
180kD
MDR1
60kD
AMPK
60kD
pAMPK^{Thr172}
60kD
Beclin1
60kD
pBeclin1^{Ser15}
60kD
p62
25kD
LC3I
LC3II
5kD
35kD
Actin

Figure3b

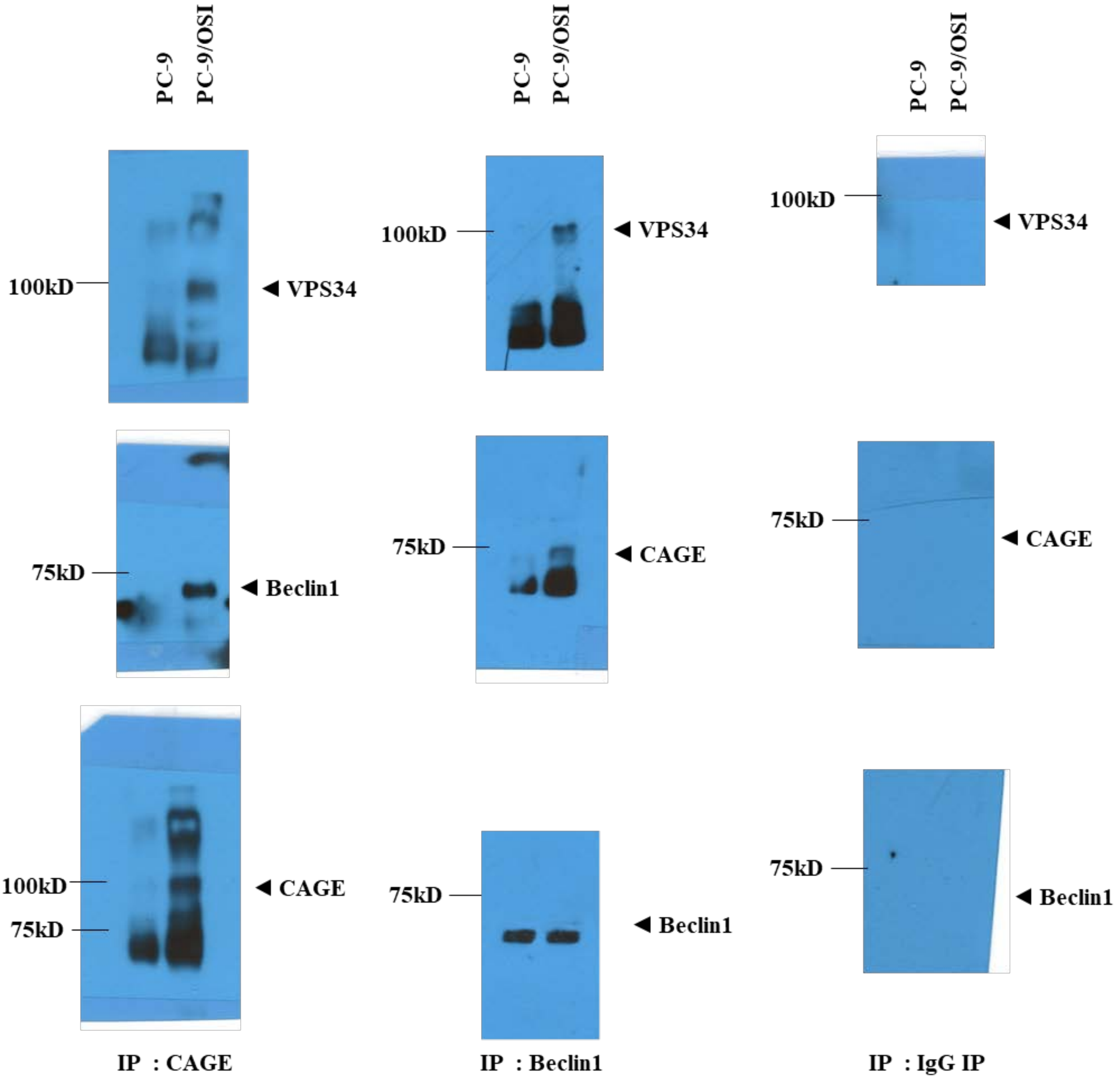
PC-9
PC-9/OSI
100kD
VPS34
75kD
Beclin1
100kD
75kD
CAGE
IP : CAGE
100kD
VPS34
75kD
CAGE
75kD
Beclin1
IP : Beclin1
100kD
VPS34
75kD
CAGE
75kD
Beclin1
IP : IgG IP

Figure3h

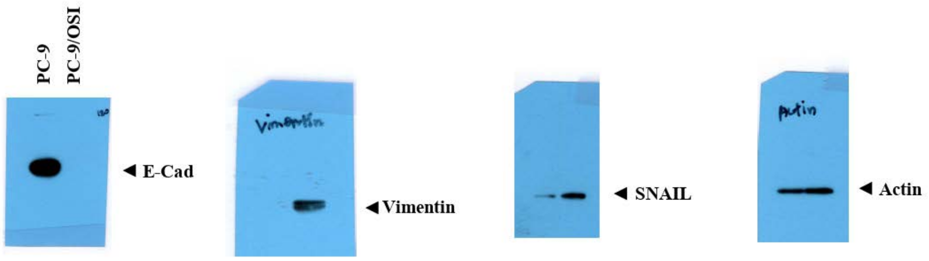

Figure4a

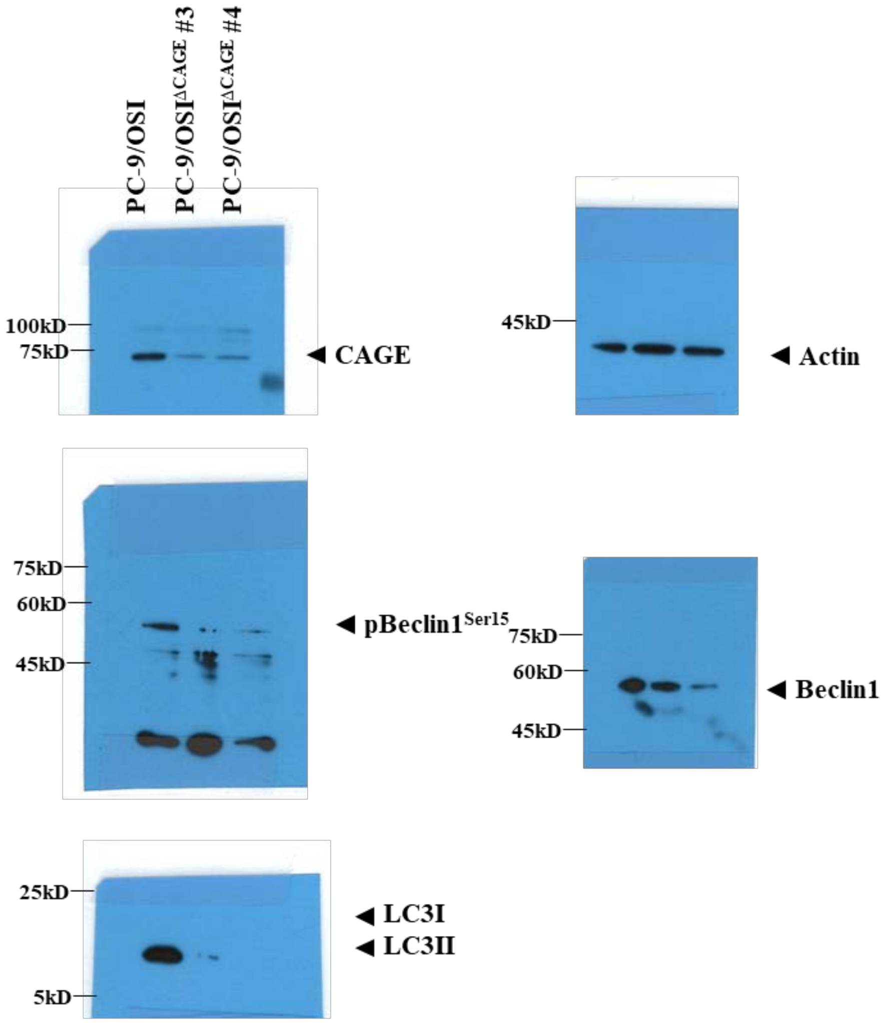

Figure4e

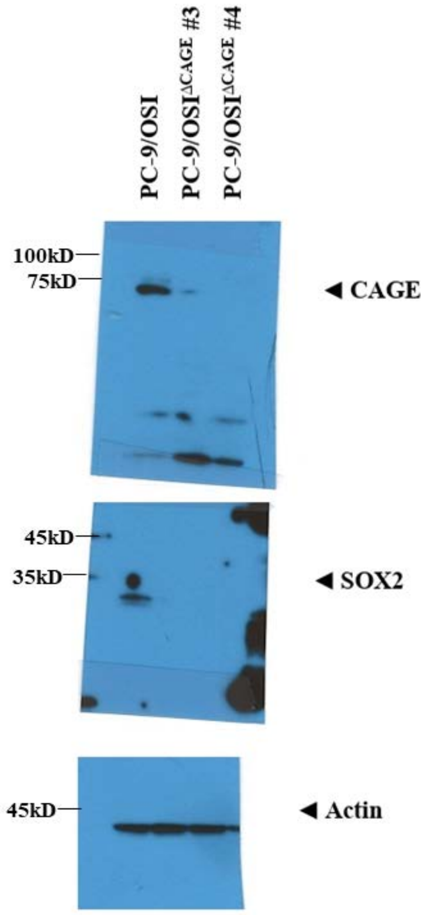

Figure4f

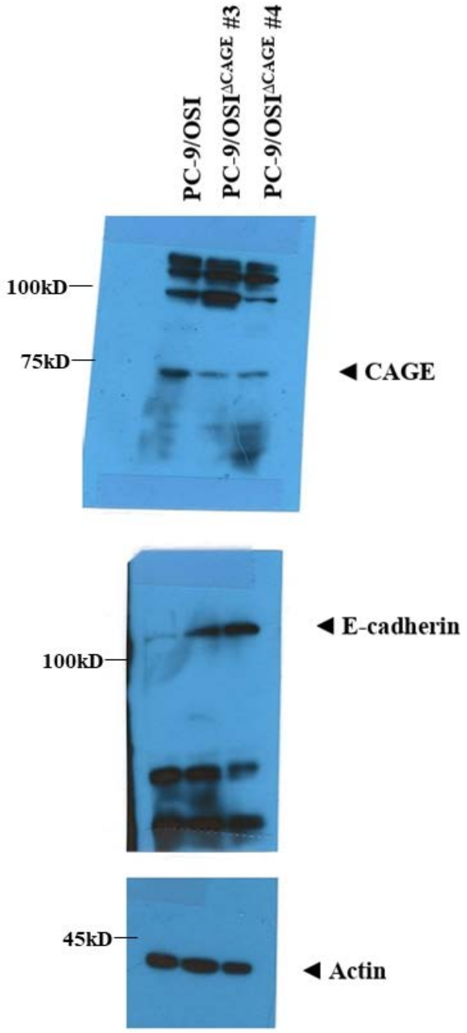

Figure5b

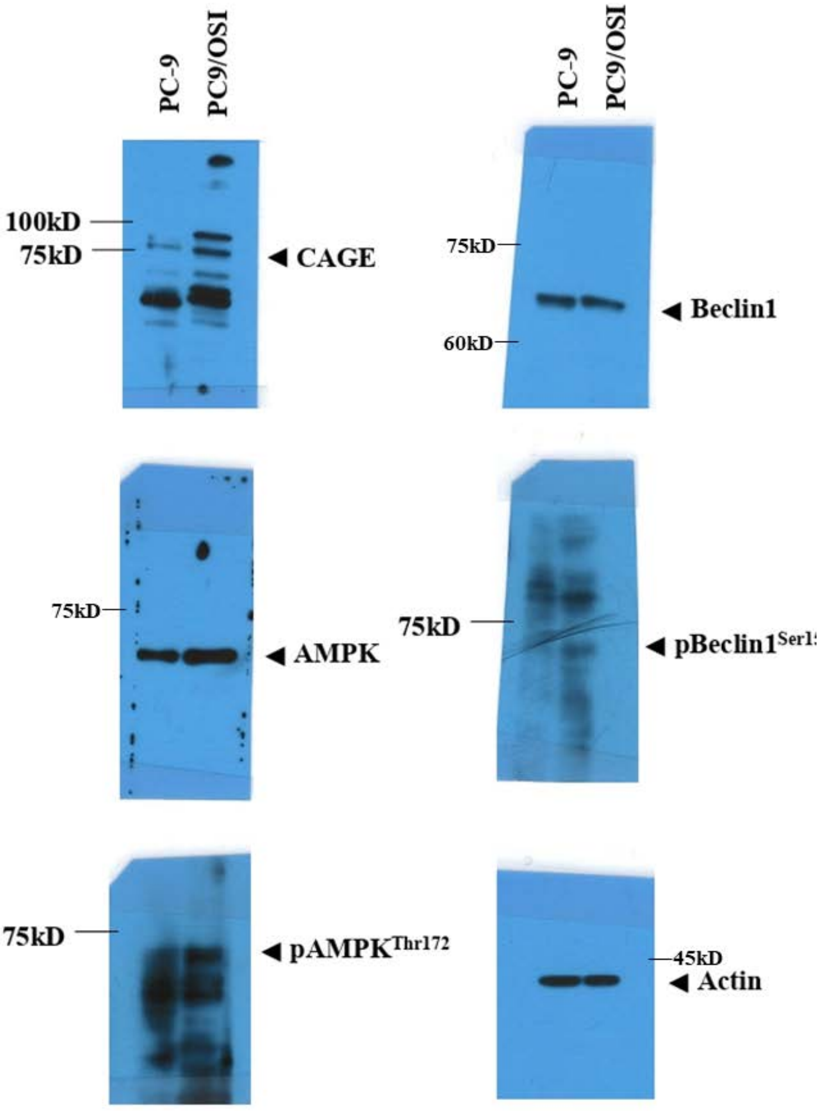

PC-9
PC9/OSI
100kD
75kD
◀ CAGE
PC-9
PC9/OSI
75kD
◀ Beclin1
60kD
75kD
◀ AMPK
75kD
◀ pBeclin1Ser1
75kD
◀ pAMPKThr172
45kD
◀ Actin

Figure5d

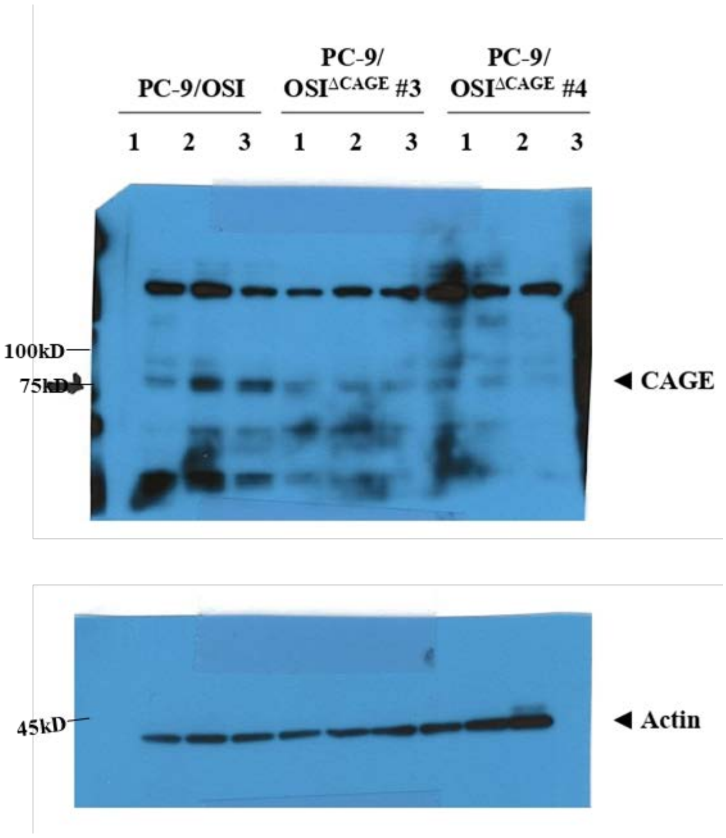
PC-9/OSI
PC-9/ OSI^ΔCAGE #3
PC-9/ OSI^ΔCAGE #4
1 2 3 1 2 3 1 2 3
100kD
75kD
◄ CAGE
45kD
◄ Actin

Supplementary Figure S2c

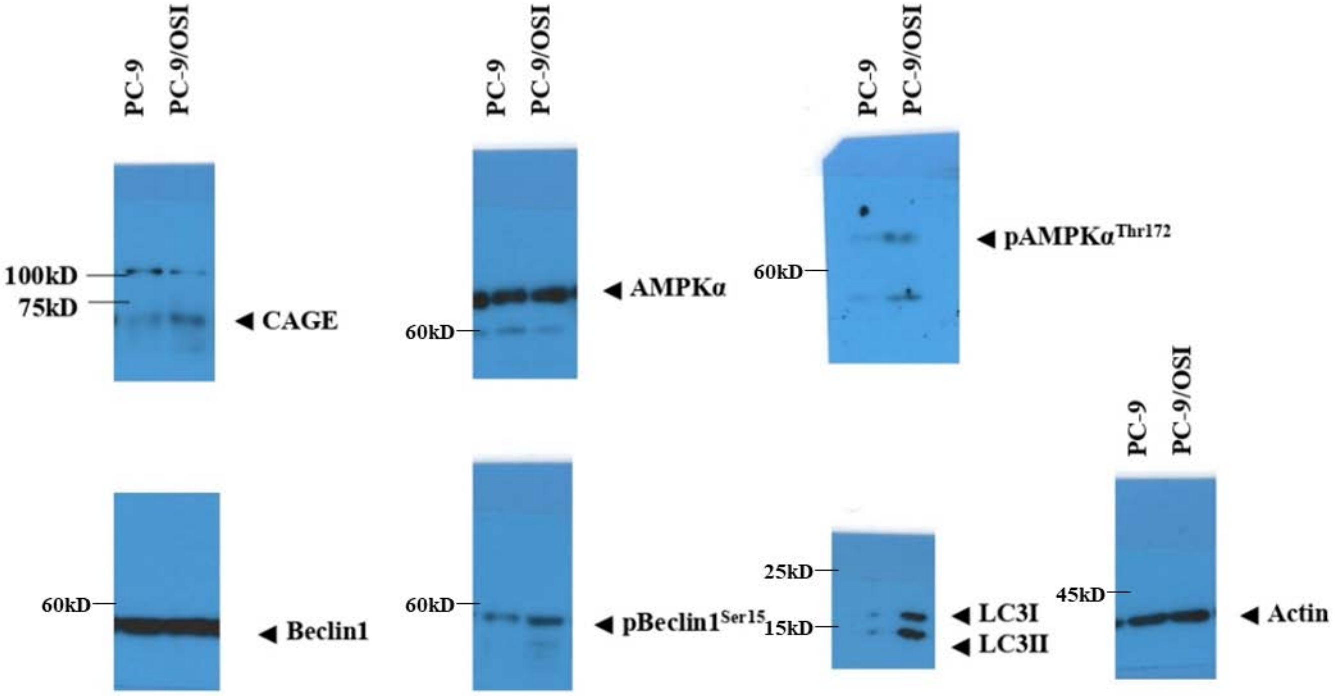

Supplementary Figure S3a

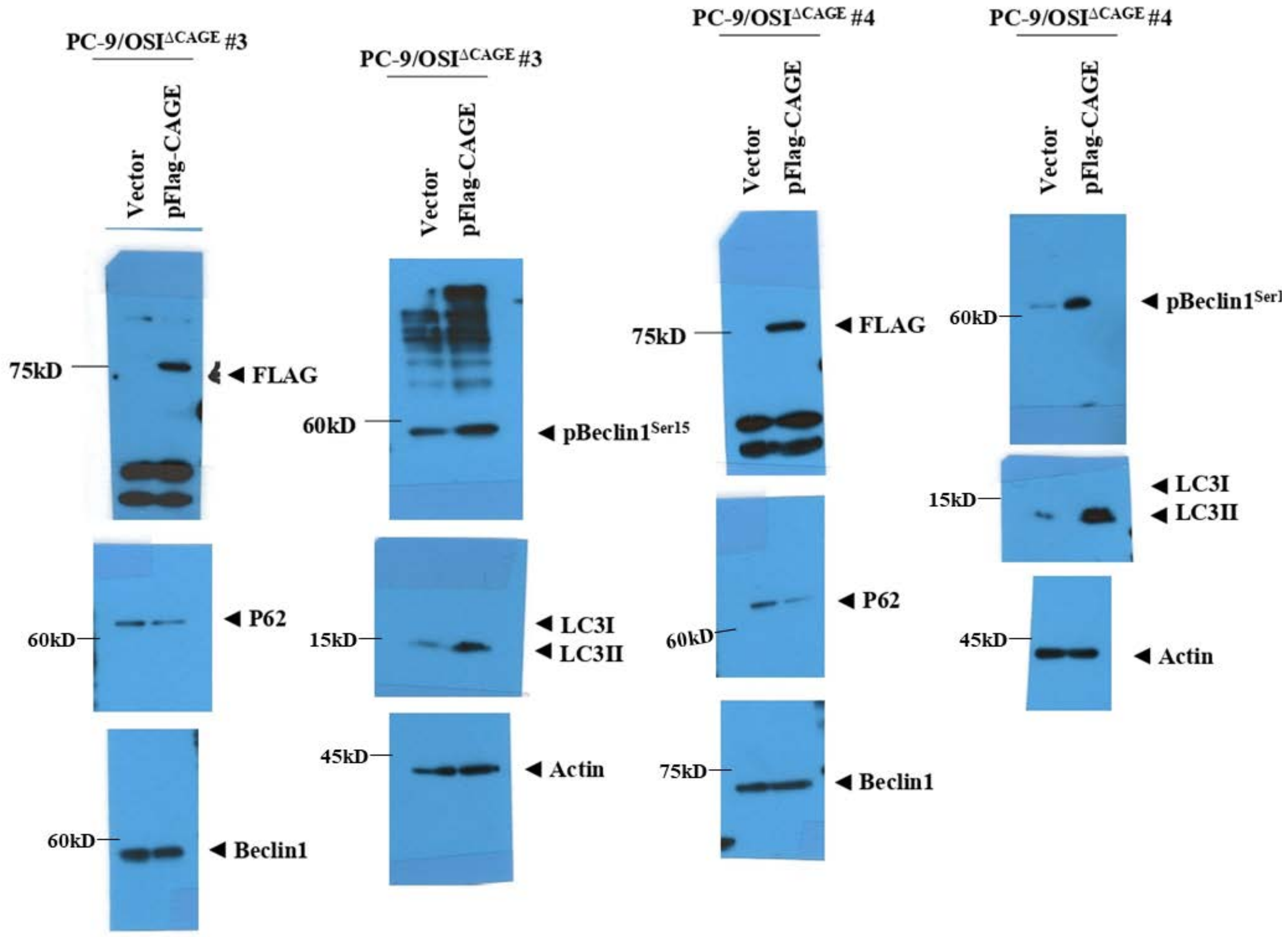

Supplementary Figure S3c

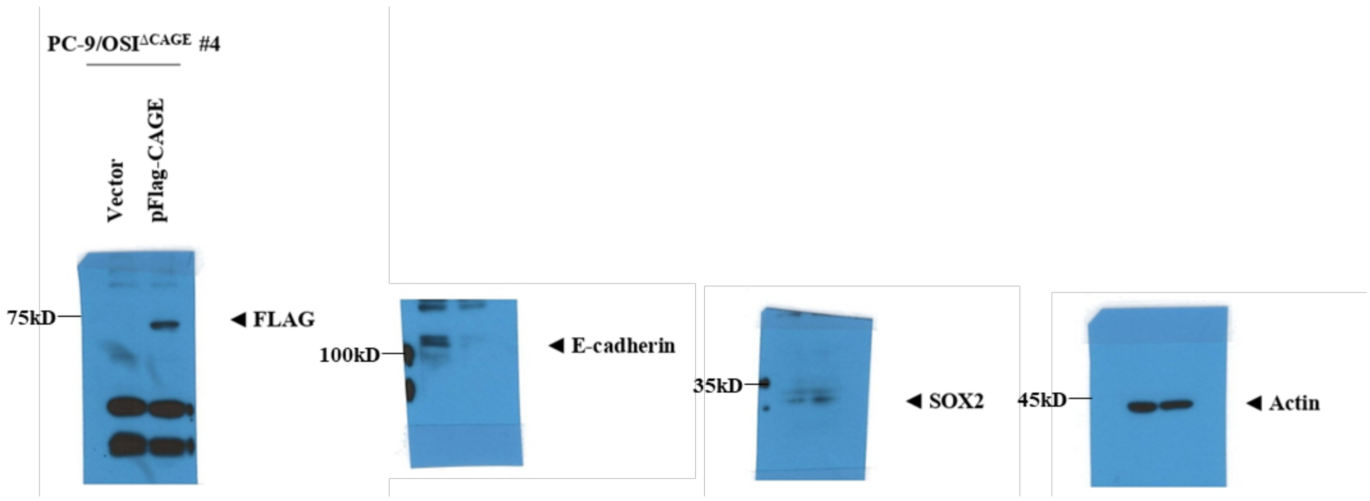

Supplementary Figure S3d

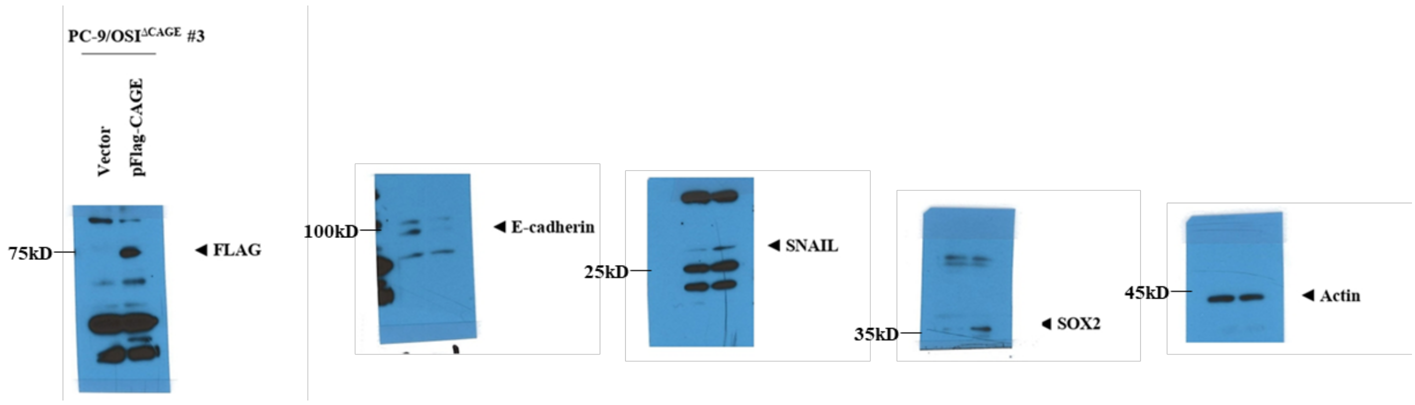

Supplement: Supplementary file 3 — Supplementary Information 3. [file 41598_2023_43124_MOESM3_ESM.pdf]
